# Endolymphatic Hydrops in Patients With Vestibular Migraine and Concurrent Meniere's Disease

**DOI:** 10.3389/fneur.2021.594481

**Published:** 2021-03-11

**Authors:** Sun-Young Oh, Marianne Dieterich, Bit Na Lee, Rainer Boegle, Jin-Ju Kang, Na-Ri Lee, Johannes Gerb, Seung-Bae Hwang, Valerie Kirsch

**Affiliations:** ^1^Department of Neurology, School of Medicine, Jeonbuk National University, Jeonju, South Korea; ^2^Research Institute of Clinical Medicine, Jeonbuk National University Hospital-Biomedical Research Institute, Jeonbuk National University, Jeonju, South Korea; ^3^Department of Neurology, University Hospital, Ludwig-Maximilians-Universität, Munich, Germany; ^4^German Center for Vertigo and Balance Disorders-IFB, University Hospital, Ludwig-Maximilians-Universität, Munich, Germany; ^5^Munich Cluster for Systems Neurology (SyNergy), Munich, Germany; ^6^Division of Oncology and Hematology, Department of Internal Medicine, Jeonbuk National University Hospital and School of Medicine, Jeonju, South Korea; ^7^Department of Radiology, Jeonbuk National University Hospital and School of Medicine, Jeonju, South Korea

**Keywords:** vestibular migraine, Meniere's disease, endolymphatic hydrops, magnetic resonance imaging, intravenous gadolinium, image analysis

## Abstract

**Objective:** Intravenous contrast agent enhanced, high-resolution magnetic resonance imaging of the inner ear (iMRI) confirmed that patients with Menière's disease (MD) and vestibular migraine (VM) could present with endolymphatic hydrops (EH). The present study aimed to investigate EH characteristics and their interrelation to neurotologic testing in patients with VM, MD, or VM with concurrent MD (VM-MD).

**Methods:** Sixty–two patients (45 females, aged 23–81 years) with definite or probable VM (*n* = 25, 19 definite), MD (*n* = 29, 17 definite), or showing characteristics of both diseases (*n* = 8) were included in this study. Diagnostic workup included neurotologic assessments including video-oculography (VOG) during caloric stimulation and head-impulse test (HIT), ocular and cervical vestibular evoked myogenic potentials (o/cVEMP), pure tone audiometry (PTA), as well as iMRI. EH's degree was assessed visually and via volumetric quantification using a probabilistic atlas-based segmentation of the bony labyrinth and volumetric local thresholding (VOLT).

**Results:** Although a relevant number of VM patients reported varying auditory symptoms (13 of 25, 52.0%), EH in VM was only observed twice. In contrast, EH in VM-MD was prevalent (2/8, 25%) and in MD frequent [23/29, 79.3%; χ^2^(2) = 29.1, *p* < 0.001, φ = 0.7]. Location and laterality of EH and neurophysiological testing classifications were highly associated (Fisher exact test, *p* < 0.005). In MD, visual semi-quantitative grading and volumetric quantification correlated highly to each other (*r*_S_ = 0.8, *p* < 0.005, two-sided) and to side differences in VOG during caloric irrigation (vestibular EH ipsilateral: *r*_S_ = 0.6, *p* < 0.05, two-sided). In VM, correlations were less pronounced. VM-MD assumed an intermediate position between VM and MD.

**Conclusion:** Cochlear and vestibular hydrops can occur in MD and VM patients with auditory symptoms; this suggests inner ear damage irrespective of the diagnosis of MD or VM. The EH grades often correlated with auditory symptoms such as hearing impairment and tinnitus. Further research is required to uncover whether migraine is one causative factor of EH or whether EH in VM patients with auditory symptoms suggests an additional pathology due to MD.

## Introduction

The Bárány Society recently published diagnostic criteria for vestibular migraine (VM) and Menière's disease (MD) (1, 2). However, their critical clinical symptoms overlap, and no specific diagnostic test can reliably distinguish them: Up to 40% of VM patients present with auditory symptoms such as aural pressure, tinnitus, and sudden sensorineural hearing loss (3, 4). Half of the MD patients present with migrainous features, such as a headache with photophobia or positive family history for migraine (5, 6). Moreover, about a quarter of VM and MD patients meet both diagnostic criteria (3, 7). Therefore, establishing the correct diagnosis remains challenging (3).

So far, the MD's pathophysiology remains unknown. Nevertheless, based on post-mortem temporal bones analyses, endolymphatic hydrops (EH) was considered a potential MD marker (8). However, EH is also known as an endpoint of different etiologies such as trauma, electrolyte imbalance (9), cellular channelopathies (10), viral infection, and autoimmune processes (11). Furthermore, fluctuating EH dependent on the time interval after a VM attack was reported recently by longitudinal MRI (12). It remains to be seen whether the EH is a bystander phenomenon or pathophysiological relevant in VM. Consequently, the EH's mere verification did not prove the desired clear-cut discriminatory diagnostic criteria between VM and MD.

The present study uses intravenous, delayed, contrast agent enhanced, high-resolution magnetic resonance imaging of the inner ear (iMRI) to investigate EH characteristics and its interrelation to neurotologic testing in patients with VM, MD, and VM with concurrent MD (VM-MD).

## Materials and Methods

### Setting and Institutional Review Board Approval

Jeonbuk National University Hospital between 2018 and 2019. Institutional review board (IRB) approval was obtained before the study's initiation (IRB No. 2017-09-022). All participants provided informed oral and written consent following the declaration of Helsinki before inclusion into the study and received monetary compensation for participation.

### Study Population

Sixty-two patients with either probable or definite VM, MD, or both disease characteristics (VM-MD) participated in this study. Diagnosis for VM was based on the consensus document of the Bárány Society and the International Headache Society (1). Diagnosis for MD was based on the classification of the Bárány Society, 2015 (2).

The classification was done based on the history (with particular attention to ear symptoms, symptoms in the head, headache, and other neurological symptoms) and the neurotological data. For example, profound hearing loss, aural fullness of one ear, or significant unilateral vestibular dysfunction (>30–35% side difference of caloric nystagmus) during or immediately after an attack pointed to MD. Migraine headache with phono- and photophobia, nausea, and vomiting indicated VM. Unfortunately, it was not always possible to differentiate between preexisting chronic auditory or vestibular signs and symptoms and acute ones during the attack.

After detailed history taking, all patients underwent neurological and neurotological testing, including 3D-video-oculography (VOG) with caloric stimulation (cVOG), video head impulse test (vHIT), cervical and ocular vestibular-evoked myogenic potentials (cVEMPs and oVEMPs, respectively), pure tone audiometry (PTA), and inner ear MRI with delayed intravenous gadolinium enhancement (iMRI).

### Nomenclature

In the following, “ipsilateral” refers to the clinically leading side and “contralateral” to the opposite side. In the case of patients presenting without a leading clinical side, a pseudorandom number generator (“Marsenne Twister” algorithm, uniform distribution) was used to generate a random number between 1 (= minimum value) and 9 (= maximum value). Even numbers meant left clinically leading side (13), and uneven numbers indicated right clinically leading side.

“Headache” pertains to the attack-associated migrainous head and neck pain or pressure, both uni- and bilateral, that fit the criteria of VM. “Auditory symptoms” comprehends attack-associated tinnitus, ear fullness, hearing loss, and or hearing fluctuation that fit the criteria for MD.

### Vestibular and Auditory Testing

#### Video-Oculography (VOG)

Eye movements and gaze stability were examined using three-dimensional VOG (3D-VOG, SMI, The Netherlands) (14). Eye movements and the ability to hold a steady gaze were evaluated during attempted fixation of visual targets located centrally or eccentrically (± 30° horizontally, ± 20° vertically). Spontaneous and gaze-evoked nystagmus, vibration-induced and head-shaking nystagmus, positional tests, horizontal saccades, and smooth pursuit eye movements were evaluated. Digitized data were analyzed using MATLAB® software.

#### VOG During Caloric Irrigation

Caloric testing with VOG was performed for both ears with 30° Celsius (C) cold and 44° C warm water. Vestibular paresis was defined as >35% asymmetry between the right- and left-sided responses; this was calculated with the formula of (15) based on the slow-phase velocity of caloric nystagmus (16):

$$\begin{array}{l} \{\left[\left(R33^{{}^{\circ}}C+R44^{{}^{\circ}}C)-(L30^{{}^{\circ}}C+L44^{{}^{\circ}}C\right)\right]/[\left(R33^{{}^{\circ}}C+R44^{{}^{\circ}}C\right)+\\ \;\;\left(L30^{{}^{\circ}}C+L44^{{}^{\circ}}C\right)]\}\times 100. \end{array}$$

#### Video Head Impulse Test (vHIT)

vHIT was performed using a video-oculography system (SLMED, Seoul, Korea). Patients were examined at a distance of 1 m from the target at eye level. The slippage of the goggles was minimized by fastening them to the head with an elastic band. Patients were seated in a height-adjustable chair, which allowed the examiner to adjust the subject's head for optimal examination. Patients were instructed to look at a point on the wall 1 m ahead. An experienced examiner conducted the examination and manually performed it more than 20 times (head rotation 15–20°, duration 150–200 ms, peak velocity > 150°/s) on both sides of each plane. Normal vHIT was defined as having a gain of ≤ 2 standard deviations (SD) of the age-matched normal gain reference range and no fixation catch-up saccades.

#### Cervical and Ocular Vestibular Evoked Myogenic Potentials

Vestibular evoked myogenic potentials (VEMPs) were evoked by air conducted (AC) 500-Hz short tone bursts (100 dB nHL; rise/fall time = 2 ms; plateau time = 1 ms; 100 trials at 5 Hz) using customized software (Cadwell Laboratories, Kennewick, WA, USA) and delivered by calibrated headphones of the same firm. Patients lay supine with surface EMG electrodes placed according to the evoked potentials [for details see (17, 18)].

For cVEMP (cervical VEMP) recordings, the recording electrode was placed over the belly of the ipsilateral sternocleidomastoid muscle, the reference electrode on the medial clavicle with self-adhesive Ag/AgCl electrodes and with the ground electrode fixed on the sternums' incisura jugularis. During unilateral stimulation, patients were asked to raise their head from the horizontal by 30° and rotate it to the contralateral side. The electromyographic (EMG) signal was amplified (bandwidth 20Hz−3kHz), sampled at 5 kHz, and then averaged for a 50 ms time window (starting from stimulus onset), for each side, respectively, and sequentially.

For oVEMP (ocular VEMP) recordings, responses obtained from the contralateral eye to the stimulated ear (the initial negative peak with short latencies around 10 ms and the following positive peak) were analyzed. The recording electrode was placed on the infraorbital ridge 1 cm below the center of each lower eyelid, the reference electrode was placed 2 cm below, and the ground electrode was on the forehead (19). During stimulation, patients were asked to fix their gaze on the target located 25° above eye level in about 60 cm distance. The EMG signal was amplified (bandwidth 10Hz−2kHz), sampled at 10 kHz, and then averaged for a 60 ms time window (starting from 2 ms before stimulus onset) for each side simultaneously.

To avoid bias due to different examiners, asymmetry ratios (AR) of VEMP amplitudes and latencies (cVEMP = p13, n23; oVEMP = n10, p15) were chosen as outcome parameters. The AR was calculated using the following formula:

$$\begin{array}{l} AR=[(larger\;response-smaller\;response)/(larger\;response+\\ \;+smaller\;response)\times 100]. \end{array}$$

#### Pure Tone Audiometry Test

Pure tone audiometry (PTA) was assessed based on the hearing levels recorded at which the subjects exhibited their worst 4-tone average during a month before Gd enhanced MRI. Medical history of acute or chronic otitis media, sudden sensorineural hearing loss, vestibular neuritis, or benign paroxysmal positional vertigo was excluded.

### Delayed Intravenous Gadolinium-Enhanced MRI of the Inner Ear (iMRI)

#### Data Acquisition

All MR imaging was acquired on a 3.0 Tesla MR scanner (Magnetom Skyra; Siemens, Erlangen, Germany) using a 64-channel phased-array head coil. All patients underwent MR imaging 4 h after administering a standard dose (0.2 ml/kg body weight, i.e., 0.1 mmol/kg body weight) of gadoterate meglumine (Gd-DOPTA, Dotarem; Guerbet). We used improved HYDROPS (HYbriD of Reversed image Of Positive endolymph signal and native image of positive perilymph Signal) imaging protocol for the evaluation of endolymphatic space previously proposed by Naganawa et al. (20) to reduce scan time. The following imaging sequences were obtained from all patients: heavily T2-weighted (hT2W) MR cisternography (MRC) for an anatomical total lymph space reference, hT2W three-dimensional (3D) fluid-attenuated inversion recovery (FLAIR) with an inversion time of 2,900 ms (positive perilymph image, PPI) and hT2W 3D FLAIR with an inversion time of 2,500 ms (positive endolymphatic image, PEI) for evaluating endolymphatic hydrops.

Detailed scan parameters were as follows. MRC acquisition parameters were: variable flip angle 3D turbo spin-echo (sampling perfection with application-optimized contrasts by using different flip angle evolutions [SPACE]); repetition time (TR), 4,400 ms; echo time (TE), 542 ms; flip angle (FA), initial refocusing FA of 180 degrees rapidly decreased to constant FA of 120 degrees for the turbo-spin-echo refocusing echo train; matrix size, 384 × 324; slices per slab, 104; slice thickness, 1 mm; field of view (FOV), 162 × 192 mm; generalized auto-calibrating partially parallel acquisition (GRAPPA) parallel imaging technique; acceleration factor, 2; number of excitations (NEX), 1.8; and scan time, 3 min 15 s. The parameters of PPI were: SPACE sequence; TR, 16,000 ms; TE, 542 ms; inversion time, 2,900 ms; FA, initial refocusing FA of 180 degree rapidly decreased to constant FA of 120 degrees for the turbo-spin-echo refocusing echo train; matrix size, 384 × 324; slices per slab, 104; slice thickness, 1 mm; FOV, 162 × 192 mm; GRAPPA parallel imaging technique with an acceleration factor of 2; NEX, 1.4; and scan time, 7 min 14 s. The PEI parameters were the same as those of PPI except for the inversion time of 2,500 ms.

HYDROPS images were generated on the scanner console by subtracting the PEI from the PPI for the visualization of endolymphatic hydrops. For the image analysis, HYDROPS-Mi2 images providing a higher contrast-to-noise ratio than HYDROPS images were also generated on a MATLAB (MATrix LABoratory) software by multiplication of HYDROPS and MRC images. Identical FOV, matrix size, and slice thickness were applied to MRC, PPI, and PEI for facilitating comparison and subtraction.

#### Semi-quantitative Visual Grading of the Endolymphatic Space

**Figure 1:**
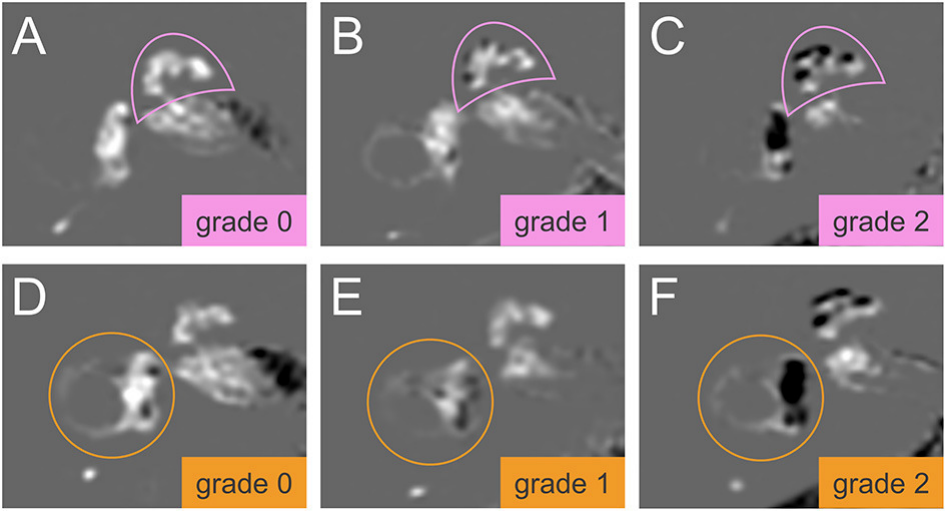
Grading of the cochlear and vestibular endolymphatic hydrops (EH). The degree of EH in cochlea (pink semicircle, top row, **A–C**) and vestibulum (orange circle, lower row, **D–F**) was classified according to Nakashima et al. (21). In HYDROPS-Mi2 images, the presence of EH as enlarged endolymphatic space (ELS) can be observed as hypointense (= dark) signal spaces surrounded by the hyperintense (= bright white) signal Gadolinium-enhanced perilymphatic space (PLS) and bony labyrinth. Grading of the cochlear EH **(A–C)** is done at the slice of the mid-modiolar level. EH in the cochlea can be categorized as grade 0 = no EH, no displacement of Reissner's membrane, contrast-enhanced PLS with no enlarged endolymphatic spaces **(A)**; grade 1 = mild, mild displacement of Reissner's membrane, ELS area (dark) of the cochlear duct is as large as the area of the scala vestibuli **(B)**; grade 2 = severe displacement of Reissner's membrane, the area of cochlear duct markedly enlarged and is larger than the area of the scala vestibuli **(C)**. Grading of the vestibular EH **(D–F)** is done at the lowest slice of the vestibulum when the lower part of the lateral semicircular canal is still visible. EH in the vestibulum can be graded as follows: Grade 0 = no vestibular EH is defined as the area ratio of the endolymphatic space to the total fluid space is not < 33.3% **(D)**; grade 1 = mild vestibular EH is defined as the area ratio exceeds 33.3% but is below 50% **(E)**; grade 2 = severe vestibular EH is defined as the area ratio exceeds 50% **(F)**. HYDROPSMi2, Hybrid of the reversed image of positive endolymph signal and native image of positive perilymph signal multiplied by T2.

An experienced neuroradiologist who was blinded to the clinical patient data classified the degree of EH in the vestibulum and cochlea according to the methods proposed by Nakashima et al. (21). The degree of EH was visually classified as none (grade 0), mild (grade 1), or severe (grade 2) in the cochlea and vestibulum separately. A detailed description with an image example of the cochlea classification and the vestibulum can be found in Figure 1. For the cochlear EH grading (Figures 1A–C), the slice with the most substantial height of the cochlear modiolus was selected. For the vestibular EH grading (Figures 1D–F), the lowest slice where the lateral semicircular canal is almost visible was chosen. The ampulla and semicircular canals were excluded from the classification process. The endolymphatic space and total fluid space (sum of the endolymphatic and perilymphatic space) of the vestibulum were outlined manually to determine the grading of vestibular EH using ImageJ software (http://rsb.info.nih.gov). The total fluid space was drawn on the MRC image, and the endolymphatic space with a negative (dark) signal was drawn on the HYDROPS-Mi2 image. The ratio of the endolymphatic space area to that of the total fluid space determined EH grading and is shown in Figures 1D–F.

#### Volumetric Quantification of the Endolymphatic Space

**Figure 2:**
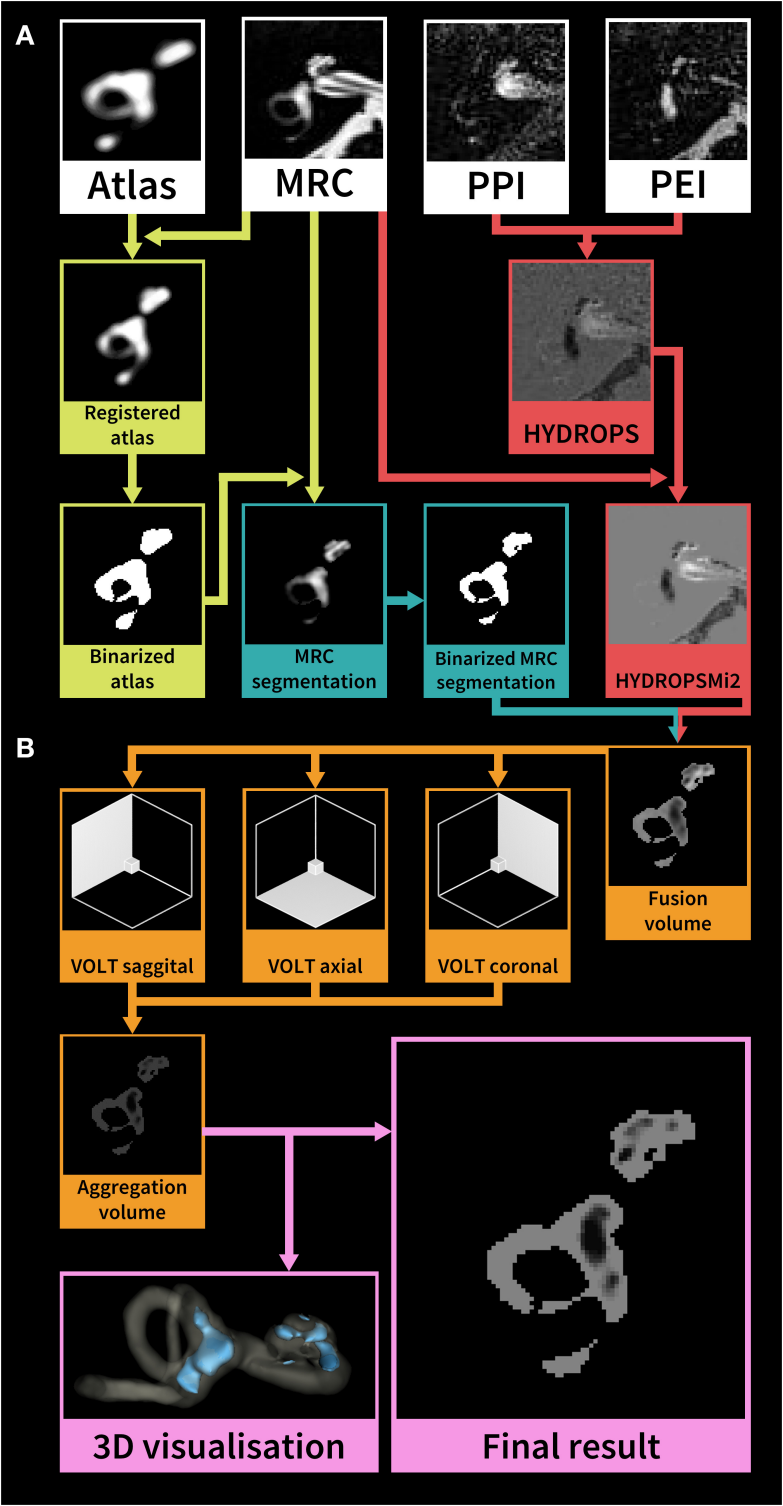
Overview of the HYDROPSMi2 volumetric quantification pipeline. The flowchart shows a step-by-step overview of an example of an exemplary single subject's right inner ear. Each color corresponds to a different processing step. The pipeline is divided into **(A)** pre-processing and **(B)** volumetric local thresholding (VOLT) processing steps: First, a probabilistic atlas of the bony labyrinth (22) is co-registered to the MRC image, binarized using 3D-thresholding and then used to segment the MRC image (green steps). Second, the HYDROPSMi2-image is created by subtracting the PEI from the PPI and multiplying the result with the MRC image (red steps). Third, a fusion volume is created from the binarized MRC-segmentation and the HYDROPSMi2-image (red and blue arrows). VOLT (23) is then performed with two algorithms and two spatial resolutions on each of the reconstructions. All VOLT-results are reconstructed into one direction and aggregated into one volume (orange steps). This aggregation volume can be used to visualize the endolymphatic space (ELS) in 3D or as a quantification of the ELS in mathematical analysis (pink steps). HYDROPSMi2, Hybrid of the reversed image of positive endolymph signal and native image of positive perilymph signal multiplied by T2; MRC, Magnetic resonance cisternography; PPI, Positive perilymph image; PEI, Positive endolymph image.

After two patients were discarded due to missing sequences (one patient with probable VM, one patient with probable MD), the data of sixty patients underwent volumetric quantification of the ELS. The method used were atlas-based segmentation of the bony labyrinth of the inner ear (22) and Volumetric Local Thresholding or VOLT (23) of the total fluid space (TFS). The following open-source software was used: 3D Slicer version 4.11 toolbox (24), ImageJ Fiji (25), and the “MorphoLibJ Toolbox” (26). An overview of the volumetric pipeline can be seen in Figure 2.

VOLT operates on a pre-segmented region-of-interest (ROI) of the inner ear, which requires a series of data pre-processing steps: *(i)* A side-independent version of the probabilistic atlas of the bony labyrinth of the inner ear (22) was created. *(ii)* A cuboid containing the subjects' inner ears (30 × 30 × 52 mm) was cropped from the MRC and HYDROPSMi2 image stacks using FiJi (27). Volumes were then converted into 8-bit and re-scaled (factor 2) using Quintic B-spline interpolation Transform-plugin (28). Also, histogram-based noise reduction was performed on the MRC-volumes to simplify atlas registration. *(iii)* Atlas registration was done through the BRAINSFIT-algorithm (29) with the following parameters: fixed image: MRC volume, moving image: atlas, mode: affine (12 DOF; degrees of freedom), percentage of samples: 0.5, maximum step length 0.5. *(iv)* MRC-images underwent segmentation into the inner ear and background. Subsequently, MRC volumes labeled as inner ear were volumetrically thresholded in three dimensions to create a binary hull. Since MRC, PPI, and PEI were precisely aligned according to the HYDROPSMi2-protocol, a dilated version of this hull could be directly applied to the HYDROPSMi2 image, creating a fusion image that could be used for direct intensity-based analysis.

Data processing with VOLT (23) on the HYDROPSMi2-fusion volumes included the following steps: First, 3D- reconstructions of the source image in three different orthogonal orientations. Second, the calculation of multiple binarized versions [two thresholding algorithms: Niblack (30), Mean; two spatial resolutions: 6, 10]. Third, aggregation into one final volume resulted in a 3D probability map, where low intensities classify as endolymph, and high intensities classify as perilymph. Fourth, the selection of cut-off 11 for endolymphatic VOLT-classifications and analysis of the resulting volumes using MorphoLibJ-Suite (26). After VOLT-processing, manual 3D cropping was performed using FiJi to differentiate between cochlear and vestibular ELS.

#### Parameters Derived From Endolymphatic Space Measures

The extent of the ELS was estimated as the mean between the ipsilateral and contralateral side in mm^3^, as well as the ratio of the ELS regarding the TFS [in %] for the complete inner ear, the cochlea (c), and the vestibulum (v). The asymmetry between the two sides was assessed using the difference (Diff) between the ipsilateral and contralateral side, the ratio regarding the TFS (Diff/TFS-Ratio in %) as well as using an asymmetry index *[(EH*_*R*_ –* EH*_*L*_*.) / (EH*_*R*_ + *EH*_*L*_*.)]* in %, again for the complete inner ear, the cochlea, and the vestibulum. Figure 2 depicts a step-by-step overview of the volumetric quantification pipeline on an exemplary single-subject level.

### Data Availability Statement

All of the individual participant data that underlie the results reported in this article will be shared after deidentification (manuscript, tables, and figures).

### Statistical Analysis

Analyses were performed using the Statistical Package for Social Sciences software (SPSS, Inc., Chicago, IL, USA). A chi-square test was used to compare univariate categorical variables, such as gender, the presence of clinical symptoms, or pathological results in diagnostic testing. Fisher's exact test was used to examine the significance of the association (contingency) between EH and neurophysiological classifications. One-way analysis of variance (ANOVA) for multiple comparisons, which was *post-hoc* Bonferroni-corrected for multiple testing, was used for scalar (volumetric quantification result, clinical diagnostic raw data) and ordinal (Semi-quantitative visual scoring result) values. Group differences were assessed between all VM vs. all MD vs. all VM-MD (A), definite VM vs. definite MD vs. all VM-MD (D), and probable VM vs. probable MD vs. all VM-MD (P). Linear agreement between parameter pairs was calculated for each method separately using the two-sided Spearman's correlation coefficient. Results were reported at a significance level of *p* < 0.05 and *p* < 0.005. Categorical values are reported as the number of cases that fit the category/number of patients in the examined group (%); ordinal or scalar values are presented as means ± standard deviations.

## Results

### Demographic and Neurotological Findings

A result overview of demographic and neurotological findings can be viewed in Table 1. Sixty-two patients were included in the current study. Twenty-five patients met the diagnostic criteria for definite or probable VM (all (a) VM: 18 females, mean age ± SD, 51.3 ± 16.0 years, range 24–84 years; 19 definite (d) VM: 14 females, 51.3 ± 16.2 years, range 23–84 years; 6 probable (p) VM: 4 females, mean age ± SD, 51.3 ± 17.1 years, range 24–70 years) and 29 patients for definite or probable MD (aMD: 19 females, 61.1 ± 9.5 years, range 38–78 years; 15 dMD: 4 females, 60.3 ± 10.0 years, range 44–78 years; 14 pMD: 11 females, 62.0 ± 9.2 years, range 38–72 years). The remaining eight patients (8 of 62 = 12.9%, 6 females, mean age ± SD, 49.0 ± 17.4 years, range 23–72 years) fulfilled the criteria for VM and MD (VM-MD; 2 fulfilling criteria for definite VM and definite MD, 4 for definite VM and probable MD, 2 for probable VM and MD) at the same time or separately in succession. VM symptoms included visual aura, photophobia with a unilateral throbbing headache; MD symptoms included vertigo with typical fluctuating aural fullness and tinnitus with documented low tone hearing impairment.

**Table 1:** Demographics and clinical features in VM, MD and VM-MD patients.

		**VM**			**MD**		**VM-MD**	
	**All**	**Definite**	**Probable**	**All**	**Definite**	**Probable**	**All**	**Statistical model**
	***n* = 25**	***n* = 19**	***n* = 6**	***n* = 29**	***n* = 15**	***n* = 14**	***n* = 8**	
Age [yrs]	51.3 ± 16.0 ▴	51.3 ± 16.2✦	51.3 ± 17.1	61.1 ± 9.5 ▴	60.3 ± 10.0✦	62.0 ± 9.2	49.0 ± 17.4 ▴,✦	[2]
Gender [females]	18/25 (72.0%)▴▴	14/19 (73.7%)✦	4/6 (66.7%)✧	19/29 (55.5%)▴▴	8/15 (53.3%)✦	11/14 (78.6%)✧	6/8 (75%)▴▴,✦,✧	[1]
Onset age of vertigo [yrs]	45.0 ± 15.6 ▴	42.9 ± 14.2✦	51.6 ± 19.3	55.0 ± 8.3 ▴	55.2 ± 9.5✦	54.8 ± 7.1	40.7 ± 15.3 ▴,✦	[2]
Duration since start of vertigo [yrs]	6.3 ± 5.5	6.7 ± 4.9	5.1 ± 7.6	6.1 ± 6.5	5.2 ± 5.3	7.2 ± 7.6	8.3 ± 7.7	
Vertigo attacks in the last 6 months	24.0 ± 37.4	28.6 ± 41.8	9.3 ± 9.8	9.6 ± 17.9	8.6 ± 16.7	10.6 ± 19.6	16.3 ± 37.1	
Headache symptoms	25/25 (100%)▴▴	19/19 (100%)✦✦	6/6 (100%)✧✧	7/29 (24.1%)▴▴	4/15 (26.7%)✦✦	5/14 (35.7%)✧✧	5/8 (62.5%)▴▴,✦✦,✧✧	[1]
Onset age of headache [yrs]	43.5 ± 16.1	41.3 ± 14.7	50.6 ± 19.6	47.8 ± 7.5	49.0 ± 9.8	47.1 ± 7.0	40.6 ± 15.2	
Duration since start of headache [yrs]	7.8 ± 6.0	8.3 ± 5.3	6.0 ± 8.1	9.2 ± 7.0	9.3 ± 4.4	9.1 ± 8.7	8.4 ± 7.7	
Auditory symptoms	9/25 (36%)	7/19 (36.8%)	2/6 (33.3%)	29/29 (100%)	15/15 (100%)	14/14 (100%)	8/8 (100%)	[1]
Tinnitus	11/25 (44%)▴	9/19 (47.4%)	2/6 (33.3%)	24/29 (82.8%)▴	14/15 (93.3%)	10/14 (71.4%)	6/8 (75.0%)▴	[1]
Bilateral	7/25 (28%)	6/19 (31.6%)	1/6 (16.7%)	4/29 (13.8%)	2/15 (13.3%)	2/14 (14.3%)	1/8 (12.5%)	
Ipsilateral	4/25 (16%)	3/19 (15.8%)	1/6 (16.7%)	20/29 (69%)	12/15 (80%)	8/14 (57.1%)	5/8 (62.5%)	
Contralateral	−	−	0/6 (0%)	0/29 (0%)	0/15 (0%)	0/14 (0%)	0/8 (0%)	
Aural fullness	3/25 (12%)▴▴	2/19 (10.5%)✦✦	1/6 (16.7%)✧✧	17/29 (58.6%)▴▴	10/15 (66.7%)✦✦	7/14 (50.0%)✧✧	6/8 (75.0%)▴▴,✦✦,✧✧	[1]
Bilateral	3/25 (12%)	2/19 (10.5%)	1/6 (16.7%)	2/29 (6.9%)	1/15 (6.7%)	1/14 (7.1%)	1/8 (12.5%)	
Ipsilateral	−	−	0/6 (0%)✧✧	14/29 (48.3%)	9/15 (60%)	5/14 (35.7%)	5/8 (62.5%)	
Contralateral	−	−	0/6 (0%)	1/29 (3.4%)	0/15 (0%)	1/14 (7.1%)	0/8 (0%)	
Aural fluctuation	3/25 (12%)▴▴	2/19 (10.5%)✦✦	1/6 (16.7%)✧✧	20/29 (69%)▴▴	14/15 (93.3%)✦✦	6/14 (42.9%)✧✧	3/8 (37.5%)▴▴,✦✦,✧✧	[1]
Bilateral	3/25 (12%)	2/19 (10.5%)	1/6 (16.7%)	−	−	−	−	
Ipsilateral	−	−	−	12/29 (41.4%)	10/15 (66.7%)	2/14 (14.3%)	2/8 (25%)	
Contralateral	−	−	−	8/29 (27.6%)	4/15 (26.7%)	4/14 (28.6%)	1/8 (12.5%)	
Hearing impairment	5/25 (20.0%)▴▴	4/19 (21.1%)✦✦	1/6 (16.7%)✧✧	25/29 (86.2%)▴▴	14/15 (93.3%)✦✦	10/14 (71.4%)✧✧	3/8 (37.5%)▴▴,✦✦,✧✧	[1]
Bilateral	−	−	−	2/29 (6.9%)	1/15 (6.7%)	1/14 (7.1%)	−	
Ipsilateral	4/25 (16%)	3/19 (15.8%)	1/6 (16.7%)	21/29 (72.4%)	13/15 (86.7%)	8/14 (57.1%)	3/8 (37.5%)	
Contralateral	1/25 (4%)	1/19 (5.3%)	−	1/29 (3.4%)	−	1/14 (7.1%)	−	
Headache and auditory symptoms	9/25 (36%)	7/19 (36.8%)	2/6 (33.3%)	9/29 (31%)	4/15 (26.7%)	5/14 (35.7%)	5/8 (62.5%)	[1]
PTA mean [dB]	21.1 ± 18.0	22.4 ± 19.0	17.5 ± 15.8	29.0 ± 18.0	34.5 ± 19.2	23.6 ± 15.5	31.6 ± 23.5	
Ipsilateral	21.0 ± 19.9	21.0 ± 19.9	15.3 ± 9.4	31.6 ± 25.2	34.8 ± 25.4	28.5 ± 25.7	17.1 ± 11.2	
Contralateral	21.2 ± 20.8	21.2 ± 20.8	19.7 ± 23.0	24.5 ± 23.5	30.2 ± 30.9	18.8 ± 11.2	46.0 ± 41.2	
Initial PTA ≥ 25 dB	6/25 (24.0%)	4/19 (21.1%)	2/6 (33.3%)	20/29 (69%)	14/15 (93.3%)	6/14 (42.9%)	2/8 (25.0%)	[1]
Low tone hearing loss pattern	3/25 (12.0%)▴▴	2/19 (10.5%)✦✦	**−**✧✧	9/29 (31%)▴▴	7/15 (46.7%)✦✦	2/14 (14.3%)✧✧	1/8 (12.5%)▴▴,✦✦,✧✧	
vHIT hVOR mean gain	0.96 ± 0.07	0.95 ± 0.06	0.99 ± 0.08	0.93 ± 0.08	0.94 ± 0.08	0.91 ± 0.07	0.98 ± 0.06	
Ipsilateral	0.96 ± 0.07	0.95 ± 0.07	0.98 ± 0.08	0.91 ± 0.11	0.93 ± 0.10	0.89 ± 0.12	0.98 ± 0.07	
Contralateral	0.96 ± 0.08	0.94 ± 0.07	1.0 ± 0.09✧	0.94 ± 0.07	0.96 ± 0.09	0.93 ± 0.04✧	0.98 ± 0.06	[2]
Abnormal vHIT hVOR gain	1/25 (4%)	1/19 (5.3%)	0/6 (0%)	5/29 (17.2%)	3/15 (20%)	2/14 (14.3%)	0/8 (0%)	
Presence of vHIT corrective saccades	−▴▴	−✦✦	**−**✧✧	6/29 (20.7%)▴▴	4/15 (26.7%)✦✦	2/14 (14.3%)✧✧	−▴▴,✦✦,✧✧	[1]
Caloric response mean [°/s]	18.6 ± 8.1	19.7 ± 8.6✦	13.8 ± 3.0	12.7 ± 7.3	11.2 ± 7.4✦	14.3 ± 7.2	13.3 ± 8.9✦	[2]
Ipsilateral	19.2 ± 10.2	20.7 ± 10.7	12.7 ± 4.5	14.8 ± 8.4	13.1 ± 8.1	16.6 ± 8.6	14.3 ± 8.8	
Contralateral	18.0 ± 8.2▴	18.7 ± 9.0✦	14.9 ± 2.0	10.5 ± 9.2▴	9.2 ± 7.9✦	11.9 ± 10.6	12.4 ± 9.7✦	[2]
Caloric response AR	18.2%	19.5%	14.3%	47.2%	47.5%	46.7%	15%	
Abnormal caloric AR ≥ 35%	1/25 (4.0%)▴▴	1/19 (5.3%)✦✦	**−**✧	17/29 (58.6%)▴▴	8/15 (53.3%)✦✦	9/14 (64.3%)✧	1/8 (12.5%)▴▴.✦✦,✧	[1]
cVEMP p13 amplitude AR ≥ 40%	1/25 (4%)	1/19 (5.3%)	−	5/29 (17.2%)	3/15 (20%)	2/14 (14.3%)	1/8 (12.5%)	
cVEMP p13 amplitude AR [%]	16.8 ± 15.0	17.7 ± 16.1	14.3 ± 12.7	16.5 ± 15.5	16.1 ± 17.2	17.1 ± 14.0	14.4 ± 18.7	
cVEMP p13 mean latency [ms]	13.8 ± 0.6	13.7 ± 0.6	14.1 ± 0.5	14.6 ± 2.6	15.0 ± 2.5	14.2 ± 2.6	14.4 ± 2.3	
Ipsilateral	13.8 ± 0.7	13.6 ± 0.7	14.1 ± 0.7	14.3 ± 2.1	14.3 ± 1.9	14.3 ± 2.4	14.3 ± 2.4	
Contralateral	13.9 ± 0.6	13.7 ± 0.7	14.1 ± 0.4	14.9 ± 3.3	15.7 ± 3.6	14.0 ± 2.9	14.5 ± 2.3	
oVEMP n10 amplitude AR ≥ 40%	4/25 (16%)	4/19 (21.1%)	−	6/29 (20.7%)	5/15 (30.3%)	1/14 (7.1%)	−	
oVEMP n10 amplitude AR [%]	25.7 ± 18.5	27.5 ± 19.9	21.3 ± 15.5	22.2 ± 18.8	27.4 ± 21.3	16.5 ± 14.5	28.8 ± 23.8	
oVEMP n10 mean latency [ms]	11.2 ± 2.1	11.4 ± 2.4	10.5 ± 0.4	11.0 ± 1.0	11.1 ± 1.0	11.0 ± 1.2	10.5 ± 0.3	
Ipsilateral	11.4 ± 2.1	11.7 ± 2.4	10.6 ± 0.4	10.7 ± 2.2	10.4 ± 2.9	11.1 ± 1.2	10.5 ± 0.4	
Contralateral	11.1 ± 2.1	11.3 ± 2.5	10.4 ± 0.5	11.0 ± 1.3	11.2 ± 1.3	10.8 ± 1.4	10.6 ± 0.4	

*▴ Chi-Square test [1], or ANOVA [2] for VM (all) vs. MD (all) vs. VM-MD (all), where p < 0.05, or ▴▴ if p < 0.005*.

*✦ Chi-Square test [1], or ANOVA [2] for VM (definite) vs. MD (definite) vs. VM-MD (all), where p < 0.05, or ✦✦ if p < 0.005*.

*✧ Chi-Square test [1], or ANOVA [2] for VM (probable) vs. MD (probable) vs. VM-MD (all), where p < 0.05, or ✧✧ if p < 0.005*.

Age between VM, MD and VM-MD group differed significantly (A: *F* = 4.6, D: *F* = 14.4, *p* < 0.05, Bonferoni-corrected) and the female proportion was higher in VM (aVM = 18/25, 72%, dVM = 14/19, 73.7%, pVM = 4/6, 66.7%) when compared to MD (aMD = 19/29, 55.5%, dMD = 8/15, 53.3%, pMD = 11/14, 78.6%) and VM-MD (6/8, 75%). The migrainous headaches in VM patients began at about 43.5 years, while their vertigo attacks began around 45 years (on average 1.5 years later). Headaches were significantly more frequent [A (=aVM vs. aMD vs. VM-MD): χ^2^(2) = 32.1, *p* < 0.001, φ = 0.7; D (=dVM vs. dMD vs. VM-MD):χ^2^(2) = 20.4, *p* < 0.001, φ =0.7, *P* (= pVM vs. pMD vs. VM-MD): χ^2^(2) = 11.1, *p* < 0.005, φ = 0.6] in VM (aVM = 25/25; dVM = 19/19; pVM 6/6, 100%) when compared to MD (aMD = 7/29, 24.1%; dMD = 4/15, 26.7%; pMD = 5/14, 35.7%) or VM-MD (5/8, 62.5%). A relevant number of patients with VM (aVM = 9 of 25, 36%; dVM = 7/19, 36.8%; pVM = 2/6, 33.3%) reported fluctuating degrees of hearing disturbances, tinnitus, or aural fullness. However, whereas ipsilateral auditory symptoms, such as tinnitus [A: χ^2^(4) = 16.3, *p* < 0.05, φ = 0.5; D: χ^2^(4) = 15.2, *p* < 0.05, φ = 0.6], aural fullness [A: χ^2^(4) = 21.6, *p* < 0.005, φ = 0.4; D:χ^2^(4) = 18.2, *p* < 0.005, φ = 0.7], aural fluctuation [A: χ^2^(4) = 21.2, *p* < 0.005, φ = 0.6; D:χ^2^(4) = 23.7, *p* < 0.005, φ = 0.8], and hearing impairment [A: χ^2^(4) = 23.5, *p* < 0.005, φ = 0.6; D:χ^2^(6) = 21.5, *p* < 0.005, φ = 0.7] were mostly observed in the MD (aMD = 21/29, 72.4%; dMD = 13/15, 86.7%; pMD = 8/14, 57.1%) and VM-MD (3/8, 37.5%) groups, the VM group (aVM = 4/25, 16%; aVM 3/15 = 15.8%; pVM = 1/6, 16.7%) presented more with bilateral auditory symptoms [A: χ^2^(2) = 12.9, *p* < 0.001, φ = 0.5; D:χ^2^(2) = 9.9, *p* < 0.001, φ = 0.5; P: χ^2^(2) = 13.29, *p* < 0.005, φ = 0.7]. Unilateral auditory symptoms in MD were accompanied with ipsilateral audio-vestibular dysfunction such as low tone hearing loss patterns, abnormal caloric asymmetry, and decreased vHIT gain with corrective saccades. Latencies and the abnormal asymmetry ratio (AR) of cervical and ocular VEMPs did not differ between groups (*p* > 0.05).

### Endolymphatic Hydrops (Visual Grading) and Its Correlation to Neurophysiological Data

An overview of the EH visual grading scores and their correlation to neurophysiological results can be viewed in Tables 2, 3, respectively. EH was significantly [A (= aVM vs. aMD vs. VM-MD):χ^2^(2) = 29.1, *p* < 0.001, φ = 0.7, D (= dVM vs. dMD vs. VM-MD):χ^2^(2) = 20.9, *p* < 0.001, φ = 0.7; P (= pVM vs. pMD vs. VM-MD): χ^2^(2) = 9.2, *p* < 0.05, φ = 0.6] more frequent in MD (all: 79.3%, definite: 80.0%, probable 71.4%) than in VM-MD (2/8, 25%) or VM (all: 8%, definite: 5.3%, probable: 16.7%). EH was observed in only two of the patients with VM (2 of 25, 8.0%); one showed mild [grade 1, following Nakashima et al. (21)], and one showed a severe [grade 2, following Nakashima et al. (21)] bilateral hydrops, either only in the cochlea or also including the vestibulum (Table 2). Both patients presented with bilateral tinnitus and aural fullness. In contrast, EH was more frequently observed ipsilaterally [A: χ^2^(4) = 15.3, *p* < 0.005, φ = 0.5; D:χ^2^(4) = 18.1, *p* < 0.005, φ = 0.7] in MD (23 of 29, 79.3%) with grade 1 (11/29, 37.9%) or grade 2 (12/29, 41.4%). VM-MD took an interim position in this respect (2/8, 25%). In addition, visual grading on the ipsilateral side differed significantly for inner ear (A: *F* = 12.8, *p* < 0.05, D: *F* = 14.9, *p* < 0.05, Bonferroni-corrected), cochlea (A: *F* = 15.8, D: *F* = 16.6, *p* < 0.05, Bonferroni-corrected), and vestibulum (A: *F* = 7.6, *p* < 0.005, D: *F* = 10.7, *p* < 0.05, Bonferroni-corrected) between VM, MD, and VM-MD (Table 2).

**Table 2:** Semi-quantitative visual scoring of endolymphatic hydrops (EH) in VM, MD, and VM-MD patients.

		**VM**			**MD**		**VM-MD**	
	**All**	**Definite**	**Probable**	**All**	**Definite**	**Probable**	**All**	**Statistical model**
	***n* = 25**	***n* = 19**	***n* = 6**	***n* = 29**	***n* = 15**	***n* = 14**	***n* = 8**	
Presence of EH	2/25 (8.0%)▴▴	1/19 (5.3%)✦✦	1/6 (16.7%)✧	23/29 (79.3%)▴▴	12/15 (80%) ✦✦	10/14 (71.4%)✧	2/8 (25.0%)▴▴, ✦✦,✧	[1]
**A) Grading**								
Grade 1 (mild)	1/25 (4.0%)	−	1/6 (16.7%)	11/29 (37.9%)	2/15 (13.3%)	8/14 (57.1%)	−	
Grade 2 (severe)	1/25 (4.0%)	1/19 (5.3%)	−	12/29 (41.4%)	10/15 (66.7%)	2/14 (14.3%)	2/8 (25.0%)	
**B) Anatomical location**								
Cochlea	2/25 (8.0%)	1/19 (5.3%)	1/6 (16.7%)	22/29 (75.9%)	9/15 (60%)	10/12 (71.4%)	2/8 (25.0%)	[1]
Ipsilateral [°]	0.08 ± 0.28▴	0.05 ± 0.23✦	0.08 ± 0.20	1.03 ± 0.87▴	1.27 ± 0.96✦	0.79 ± 0.70✧	0.25 ± 0.46▴,✦	[2]
Contralateral [°]	0.12 ± 0.40	0.11 ± 0.46	0.08 ± 0.20	0.21 ± 0.41	0.20 ± 0.41	0.21 ± 0.43	−	
Vestibulum	1/25 (4.0%)▴▴	1/19 (5.3%)✦	0/6 (0%)✧✧	13/29 (44.8%)▴▴	12/15 (80%)✦	4/14 (21.4%)✧✧	1/8 (12.5%)▴▴,✦,✧✧	[1]
Ipsilateral [°]	0.08 ± 0.40 ▴	0.11 ± 0.46✦	0.17 ± 0.41	0.79 ± 0.94 ▴	1.13 ± 0.99✦	0.43 ± 0.76	0.13 ± 0.35 ▴,✦	[2]
Contralateral [°]	0.08 ± 0.40	0.11 ± 0.46	0.17 ± 0.41	−	−	−	−	
Inner ear (cochlea & vestibulum)	1/25 (4.0%)	1/19 (5.3%)	−	12/29 (41.4%)	9/15 (60%)	3/14 (21.4%)	1/8 (12.5%)	[1]
Ipsilateral [°]	0.08 ± 0.31 ▴	0.08 ± 0.34✦	−	0.91 ± 0.85▴	1.20 ± 0.92✦	0.61 ± 0.66	0.19 ± 0.37▴,✦	[2]
Contralateral [°]	0.10 ± 0.41	0.11 ± 0.46	−	0.10 ± 0.21	0.10 ± 0.21	0.11 ± 0.21	−	
**C) Laterality of EH**								
Bilateral	2/25 (8.0%)▴▴	1/19 (5.3%)✦✦	1/6 (16.7%)	3/29 (10.3%)▴▴	1/15 (16.7%)✦✦	2/14 (14.3%)	−▴▴,✦✦	[1]
Unilateral	−▴▴	−✦✦	−	19/29 (65.5%)▴▴	11/15 (73.7%)✦✦	8/14 (57.1%)	2/8 (25.0%)▴▴,✦✦	
Ipsilateral	−▴▴	−✦✦	−	16/29 (55.2%)▴▴	9/15 (60%)✦✦	7/14 (50%)	−▴▴,✦✦	
Contralateral	−▴▴	−✦✦	−	3/29 (10.3%)▴▴	2/15 (13.3%)✦✦	1/14 (7.1%)	−▴▴,✦✦	

*▴ Chi-Square test [1], or ANOVA [2] for VM (all) vs. MD (all) vs. VM-MD (all), where p < 0.05, or ▴▴ if p < 0.005*.

*✦ Chi-Square test [1], or ANOVA [2] for VM (definite) vs. MD (definite) vs. VM-MD (all), where p < 0.05, or ✦✦ if p < 0.005*.

*✧ Chi-Square test [1], or ANOVA [2] for VM (probable) vs. MD (probable) vs. VM-MD (all), where p < 0.05*.

**Table 3:** Interrelation between EH characteristics and neurophysiological testing results.

	**Neurophysiological testing results**					
**Location of EH**	**Normal *n* = 24**	**Vestibular deficits *n* = 13**	**Cochlear deficits *n* = 12**	**Vestibulocochlear deficits *n* = 13**	**Total *n* = 62**	***Fisher's exact test***
**A) Anatomical location of EH and correlation to neurophysiological data based on visual grading**						
No EH	19 (79.2%)	8 (61.5%)	6 (50.0%)	2 (15.4%)	35	
Vestibular EH	0 (0%)	1 (7.7%)	0 (0%)	0 (0%)	1	*p < 0.005*
Cochlear EH	4 (16.7%)	2 (15.4%)	2 (16.7%)	4 (30.8%)	12	
Vestibular and cochlear EH	1 (4.2%)	2 (15.4%)	4 (33.3%)	7 (53.8%)	14	
	**Neurophysiological testing results**					
**Laterality of EH**	**Normal** ***n*** **=** **28**	**Right-sided deficits** ***n*** **=** **12**	**Left-sided deficits** ***n*** **=** **16**	**Bilateral deficits** ***n*** **=** **6**	**Total** ***n*** **=** **62**	***Fisher's exact test***
**B) Laterality of EH and correlation to neurophysiological data based on visual grading**						
No EH	22 (78.6%)	3 (25.0%)	5 (31.3%)	4 (66.7%)	34	
Right-sided EH	4 (14.3%)	8 (66.7%)	1 (6.3%)	1 (16.7%)	14	*p < 0.005*
Left-sided EH	0 (0%)	0 (0%)	9 (56.3%)	1 (16.7%)	10	
Bilateral EH	2 (7.1%)	1 (8.3%)	1 (6.3%)	0 (0%)	4	

The main common feature seemed to be auditory symptoms that correlated with initial PTA > 25 dB (*r*_S_ = 0.3, *p* < 0.05, two-sided) and low tone hearing loss pattern (*r*_S_ = 0.4, *p* < 0.005, two-sided). Matching this, the visual EH grading of the ipsilateral cochlea significantly correlated with the level of PTA thresholds (*r*_S_ = 0.4, *p* < 0.005, two-sided) and VOG during caloric irrigation (A: *r*_S_ = 0.5, *p* < 0.005, two-sided) on the ipsilateral side, as well as side difference (SD) during caloric irrigation (D: *r*_S_ = 0.7, *p* < 0.05, two-sided). The EH's anatomical location (cochlea, vestibulum, both) and how they correlated with the respective dysfunction (e.g., PTA for cochlea, caloric asymmetry, vHIT) is presented in Table 3.

Overall pathologies, patients with cochlear deficits had cochlear EH, and those with vestibular deficits had vestibular EH (Fisher's exact test, *p* < 0.005, Table 3A). Furthermore, the laterality of EH correlated positively with the lesion side of vestibular and/or cochlear deficits (Fisher's exact test, *p* < 0.005, Table 3B).

### Volumetric Quantification of the ELS and Its Correlation to Neurophysiological Data

An overview of the results can be seen in Table 4. Overall, the patient groups' characteristics were most pronounced for definite diagnoses, followed by all (definite and probable) and least pronounced between patients with probable diagnoses. Two interrelations were analyzed: Parameters referring to the sheer size of the ELS (mean ELS, ELS/TFS-Ratio) and parameters referring to the symmetry between ipsilateral and contralateral inner ear (difference of the ELS between the sides (Diff), Diff/TFS-Ratio, as well as the asymmetry-index or AI).

**Table 4:** Volumetric quantification results for VM, MD, and VM-MD patients.

		**VM**			**MD**		**VM-MD**
	**All**	**Definite**	**Probable**	**All**	**Definite**	**Probable**	**All**
	***n* = 24**	***n* = 19**	***n* = 5**	***n* = 28**	***n* = 15**	***n* = 13**	***n* = 8**
**Inner ear**							
ELS mean [mm^3^]	10.1 ± 4.3 (3.8–21.3)	10.3 ± 4.8 (3.8–21.3)	9.6 ± 1.9 (7.8–12.2)	10.8 ± 5.2 (3.8–25.0)	12.7 ± 5.8 (4.3–25.0)	8.7 ± 3.5 (3.8–15.2)	9.5 ± 3.8 (3.8–15.3)
Ipsilateral	10.2 ± 4.4 (3.6–21.4)	10.4 ± 4.9 (3.6–21.4)✦	9.6 ± 2.3 (5.8–11.7)	13.1 ± 8.3 (3.7–36.5)	16.7 ± 9.7 (3.7–36.5)✦	9.1 ± 3.4 (4.3–13.9)	9.2 ± 5.2 (3.4–19.2)✦
Contralateral	10.1 ± 4.6 (1.5–21.3)	10.2 ± 5.0 (1.5–21.3)	9.5 ± 2.3 (6.6–12.8)	8.5 ± 3.6 (1.9–16.9)	8.7 ± 3.2 (4.9–15.6)	8.4 ± 4.1 (1.9–16.9)	9.8 ± 2.9 (4.2–14.2)
ELS/TFS–Ratio [%]	4.1 ± 1.5 (1.7–7.8)	4.1 ± 1.7 (1.7–7.8)	3.9 ± 0.6 (3.3–4.8)	4.4 ± 2.0 (1.7–9.5)	5.2 ± 2.2 (1.9–9.5)	3.5 ± 1.4 (1.7–6.1)	3.9 ± 1.5 (1.6–6.3)
Ipsilateral	4.1 ± 1.6 (1.6–7.9)	4.2 ± 1.8 (1.6–7.9)✦	3.9 ± 0.9 (2.6–4.7)	5.3 ± 3.3 (1.7–15.0)	6.8 ± 3.8 (1.7–15.0)✦	3.6 ± 1.3 (1.9–5.5)	3.8 ± 2.1 (1.5–7.9)✦
Contralateral	4.0 ± 1.6 (0.6–7.7)	4.1 ± 1.8 (0.6–7.7)	3.8 ± 0.8 (2.8–4.9)	3.5 ± 1.4 (0.8–6.7)	3.6 ± 1.3 (2.1–6.0)	3.3 ± 1.6 (0.8–6.7)	4.0 ± 1.1 (1.8–5.7)
Diff [mm^3^]	1.9 ± 1.6 (0.1–6.8)▴	1.8 ± 1.7 (0.1–6.8)✦	2.2 ± 1.2 (0.9–4.0)	5.7 ± 6.8 (0.2–27.9)▴	8.6 ± 8.1 (0.2–27.9)✦	2.3 ± 1.6 (0.3–4.8)	2.6 ± 2.4 (0.6–36.2)✦
Diff–Ratio [%]	0.8 ± 0.7 (0–3.0)▴	0.7 ± 0.7 (0–3.0)✦	0.9 ± 0.6 (0.3–1.7)	2.3 ± 2.8 (0.1–12.0)▴	3.5 ± 3.4 (0.1–12.0)✦	0.9 ± 0.7 (0.1–1.9)	1.1 ± 1.0 (0.2–3.2)▴,✦
Asymmetry–Index [%]	11.6 ± 12.5 (0.4–61.0)▴	11.3 ± 13.5 (0.4–61.0)✦	12.7 ± 8.7 (4.4–25.5)	22.8 ± 18.3 (0.9–62.1)▴	29.0 ± 19.2 (0.9–62.1)✦	15.8 ± 15.0 (1.5–51.2)	14.2 ± 10.2 (2.7–26.1)✦
TFS	245.5 ± 21.8	245.0 ± 21.2	247.5 ± 26.8	245.4 ± 15.1	241.2 ± 12.7	250.1 ± 13.3	244.3 ± 9.5
	(212.2–290.6)	(212.2–281.8)	(222.6–290.6)	(202.0–269.9)	(229.4–265.4)	(229.7–269.9)	(232.7–261.6)
**Cochlea**							
cELS mean [mm^3^]	2.4 ± 1.0 (0.8–4.5)	2.4 ± 1.0 (0.8–4.5)✦	2.2 ± 0.6 (1.7–3.0)	2.7 ± 2.1 (0.1–7.9)	3.5 ± 2.4 (0.6–7.9)✦	1.7 ± 1.1 (0.1–3.8)	1.6 ± 1.7 (0.1–4.0)✦
Ipsilateral	2.4 ± 1.2 (0.5–5.2)	2.5 ± 1.3 (0.5–5.2)✦	2.0 ± 0.6 (1.5–3.1)	3.4 ± 3.4 (0.1–12.2)	4.8 ± 4.0 (0.2–12.2)✦	1.8 ± 1.3 (0.1–4.3)	1.9 ± 1.9 (0.2–6.2)✦
Contralateral	2.4 ± 1.1 (0.1–4.5)	2.4 ± 1.1 (0.1–4.5)	2.4 ± 1.1 (1.6–4.1)	2.0 ± 1.2 (0.03–5.5)	2.3 ± 1.1 (0.7–5.5)	1.5 ± 1.1 (0.03–4.0)	1.4 ± 0.9 (0–2.4)
cELS/cTFS–Ratio [%]	3.2 ± 1.2 (1.1–6.1)	3.2 ± 1.3 (1.1–6.1)✦	2.9 ± 0.8 (2.1–3.8)	3.5 ± 2.6 (0.1–10.2)	4.7 ± 2.9 (0.9–10.2)✦	2.2 ± 1.4 (0.1–4.9)	2.2 ± 1.5 (0.1–4.9)✦
Ipsilateral	3.1 ± 1.5 (0.8–7.1)	3.3 ± 1.7 (0.8–7.1)✦	2.6 ± 0.8 (1.9–3.8)	4.4 ± 4.2 (0.1–15.5)	6.2 ± 5.0 (0.3–15.5)✦	2.4 ± 1.6 (0.1–5.1)	2.6 ± 2.3 (0.2–7.5)✦
Contralateral	3.2 ± 1.5 (0.1–18.2)	3.2 ± 1.5 (0.1–6.3)	3.1 ± 1.6 (1.9–5.7)	2.6 ± 1.5 (0.04–7.1)	3.1 ± 1.4 (1.1–7.1)	2.0 ± 1.5 (0.04–5.1)	1.9 ± 1.2 (0–3.4)
cDiff [mm^3^]	0.9 ± 0.9 (0.3–2.8)	0.9 ± 0.8 (0–2.8)✦	0.7 ± 1.1 (0.1–2.6)	1.8 ± 2.6 (0–10.2)	2.8 ± 3.2 (0.1–10.2)✦	0.6 ± 0.7 (0–2.4)	1.2 ± 1.4 (0.2–4.3)✦
cDiff–Ratio [%]	1.2 ± 1.1 (0–3.9)	1.2 ± 1.1 (0–3.9)✦	1.0 ± 1.5 (0.1–3.6)	2.3 ± 3.4 (0–13.2)	3.7 ± 4.2 (0.1–13.2)✦	0.7 ± 0.8 (0–2.9)	1.6 ± 1.7 (0.2–5.3)✦
cAsymmetry–Index [%]	21.9 ± 21.9 (0–89.3)	23.0 ± 22.6 (0–89.3)	14.3 ± 19.6 (1.0–25.2)	27.5 ± 21.7 (0.6–70.8)	33.0 ± 25.2 (2.1–70.8)	21.2 ± 15.0 (0.6–52.6)	41.1 ± 30.0 (13.1–100.0)
cTFS	75.1 ± 5.3	74.0 ± 4.4	79.2 ± 7.2	74.8 ± 5.7	73.5 ± 6.2	76.3 ± 5.0	72.6 ± 5.7
	(67.1–86.9)	(67.1–80.4)	(70.0–87.0)	(62.2–84.2)	(62.2–83.6)	(69.7–84.2)	(65.2–81.0)
**Vestibulum**							
vELS mean [mm^3^]	7.8 ± 4.0 (2.2–19.3)	7.9 ± 4.3 (2.2–19.3)	7.3 ± 2.4 (4.9–10.6)	8.2 ± 3.6 (2.4–17.4)	9.2 ± 3.8 (2.4–17.4)	7.0 ± 3.1 (2.6–12.6)	7.9 ± 3.1 (2.6–11.5)
Ipsilateral	7.8 ± 4.1 (2.3–20.3)	7.9 ± 4.5 (2.3–20.3)✦	7.6 ± 2.3 (4.4–9.9)	9.8 ± 5.3 (2.5–24.2)	12.0 ± 6.0 (2.5–24.2)✦	7.2 ± 2.9 (3.9–12.6)	7.3 ± 3.7 (2.1–13.0)✦
Contralateral	7.7 ± 4.1 (1.4–18.2)	7.8 ± 4.4 (1.4–18.2)	7.1 ± 2.8 (3.6–11.2)	8.5 ± 3.6 (1.9–16.9)	6.3 ± 3.2 (2.4–12.3)	6.8 ± 3.7 (1.3–14.3)	8.4 ± 2.7 (3.2–11.8)
vELS/vTFS–Ratio [%]	4.5 ± 2.0 (1.4–10.0)	4.5 ± 2.2 (1.4–10.0)	4.3 ±1.2 (3.1–6.1)	4.8 ± 2.1 (1.5–9.7)	5.4 ± 2.2 (1.5–9.7)	4.0 ± 1.7 (1.7–7.2)	4.6 ± 1.8 (1.5–7.0)
Ipsilateral	4.6 ± 2.1 (1.6–10.8)	4.6 ± 2.4 (1.6–10.8)✦	4.6 ± 1.2 (2.9–5.7)	5.7 ± 3.1 (1.6–14.8)	7.0 ± 3.4 (1.6–14.8)✦	4.2 ± 1.6 (2.5–7.3)	4.3 ± 2.3 (1.3–8.2)✦
Contralateral	4.4 ± 2.1 (0.9–9.2)	4.5 ± 2.3 (0.9–9.2)	4.2 ± 1.5 (2.4–6.5)	3.5 ± 1.4 (0.8–6.7)	3.8 ± 1.9 (1.4–7.1)	3.9 ± 2.1 (0.8–8.1)	4.9 ± 1.6 (1.8–7.0)
vDiff [mm^3^]	1.7 ± 1.2 (0.3–5.5)▴	1.7 ± 1.3 (0.3–5.5)✦	1.6 ± 0.5 (1.1–2.5)	4.4 ± 4.3 (0–17.8)▴	6.3 ± 5.1 (0.1–17.8)✦	2.3 ± 1.6 (0–5.7)	1.9 ± 1.7 (0.2–4.1)✦
vDiff–Ratio [%]	1.0 ± 0.8 (0.2–3.6)▴	1.0 ± 0.8 (0.2–3.6)✦	1.0 ± 0.4 (0.5–1.6)	2.6 ± 2.6 (0–11.4)▴	3.7 ± 3.1 (0–11.4)✦	1.3 ± 0.9 (0–3.2)	1.1 ± 1.0 (0.1–2.5)✦
vAsymmetry–Index [%]	13.0 ± 11.5 (2.6–53.3)▴	13.1 ± 12.4 (2.6–53.3)✦	12.5 ± 7.9 (0.9–4.0)	25.0 ± 19.3 (0–59.8)▴	31.1 ± 20.8 (1.2–59.8)✦	17.9 ± 15.1 (0–50.6)	14.2 ± 12.4 (0.9–36.2)✦
vTFS	170.4 ± 3.9	171.0 ± 18.2	168.3 ± 21.4	170.6 ± 11.0	167.8 ± 10.7	173.8 ± 10.8	171.7 ± 8.3
	(212.2–290.6)	(145.2–203.3)	(152.7–203.6)	(139.8–186.2)	(139.8–183.8)	(152.7–186.2)	(161.3–185.3)

*▴ ANOVA Bonferroni-corrected for multiple comparisons for VM (all) vs. MD (all) vs. VM-MD (all), where p < 0.05*.

*✦ ANOVA Bonferroni-corrected for multiple comparisons for VM (definite) vs. MD (definite) vs. VM-MD (all), where p < 0.05, or ✦✦ if p < 0.005*.

*✧ ANOVA Bonferroni-corrected for multiple comparisons for VM (probable) vs. MD (probable) vs. VM-MD (all) showed no significant differences*.

Mean ELS were most prominent on the ipsilateral side in definite MD (inner ear: 16.7 ± 9.7 mm^3^, cochlea: 4.8 ± 4.0 mm^3^, vestibulum: 12.0 ± 6.0 mm^3^), while definite VM (inner ear: 10.4 ± 4.9 mm^3^, cochlea: 2.5 ± 1.3 mm^3^, vestibulum: 7.9 ± 4.5 mm^3^), and VM-MD (inner ear: 9.5 ± 3.8 mm^3^, cochlea: 1.9 ± 1.9 mm^3^, vestibulum: 7.3 ± 3.7 mm^3^) were less pronounced (*F* = 4.1, *p* < 0.05, Bonferroni-corrected). In addition, ELS/TFS-Ratio on the ipsilateral side differed significantly (*F* = 4.9, *p* < 0.05, Bonferroni-corrected) for definite VM (4.2 ± 1.8%) vs. definite MD (6.8 ± 3.8%) vs. VM-MD (3.8 ± 2.1%). Nonetheless, differences between groups were most pronounced in the parameters referring to the asymmetry of the ipsilateral and contralateral ELS, such as the asymmetry index (AI: *F* = 3.7, cAI: *F* = 4.1, vAI: *F* = 4.1, *p* < 0.05, Bonferroni-corrected) and difference (Diff: *F* = 8.0, cDiff: *F* = 3.6, vDiff *F* = 9.3, *p* < 0.05, Bonferroni-corrected) between VM (Diff: 1.8 ± 6.3 mm^3^, cDiff: 0.9 ± 0.9 mm^3^, vDiff: 1.7 ± 1.3 mm^3^) and MD (Diff: 8.6 ± 8.1 mm^3^, cDiff: 4.7 ± 2.9 mm^3^, vDiff: 6.3 ± 5.1 mm^3^), and less when compared to VM-MD (Diff: 2.6 ± 2.4 mm^3^, cDiff: 1.2 ± 1.4 mm^3^, vDiff: 1.9 ± 1.0 mm^3^).

In MD, volumetric quantification parameters correlated highly with the semi-quantitative visual grading of the EH. In particular, the parameters referring to the absolute size of the ELS (mean, ipsilateral) correlated highly with the visual grade of the cochlea (mean: *r*_S_ = 0.9, ipsilateral: *r*_S_ = 0.8, *p* < 0.005, two-sided), vestibulum (ipsilateral: *r*_S_ = 0.8, *p* < 0.005, two-sided), and the whole inner ear (mean: *r*_S_ = 0.7, ipsilateral: *r*_S_ = 0.8, *p* < 0.005, two-sided). The highest correlations were seen with parameters referring to the “ELS side relation” in the cochlea (AI: *r*_S_ = 0.7, Diff: *r*_S_ = 0.8, Diff/TFS-Ratio: *r*_S_ = 0.8, *p* < 0.005, two-sided) vestibulum (AI: *r*_S_ = 0.8, Diff: *r*_S_ = 0.9, Diff/TFS-Ratio: *r*_S_ = 0.9, *p* < 0.005, two-sided), or inner ear (AI: *r*_S_ = 0.7, Diff: *r*_S_ = 0.8, Diff/TFS-Ratio: *r*_S_ = 0.8, *p* < 0.005, two-sided). Parameters referring to the “ELS side relation” were the ELS difference between the ipsilateral and contralateral side, the ELS/TFS-Ratio of said difference, as well as the asymmetry index. In addition, quantitative ELS measurements in definite MD correlated with the side difference in VOG during caloric irrigation (ELS mean: *r*_S_ = 0.7, ELS/TFS: *r*_S_ = 0.6, ELS ipsilateral: *r*_S_ = 0.7, cELS mean: *r*_S_ = 0.8, cELS ipsilateral: *r*_S_ = 0.7, cELS/cTFS: *r*_S_ = 0.9, vELS mean: *r*_S_ = 0.6, vELS ipsilateral: *r*_S_ = 0.6, vELS/vTFS: *r*_S_ = 0.5, *p* < 0.05, two-sided), as well as with low tone hearing loss patterns in PTA (ELS mean: *r*_S_ = 0.6, ELS ipsilateral: *r*_S_ = 0.7, ELS/TFS: *r*_S_ = 0.7, AI: *r*_S_ = 0.7, Diff: *r*_S_ = 0.6, Diff/TFS-Ratio: *r*_S_ = 0.7, vELS ipsilateral: *r*_S_ = 0.8, vELS/vTFS: *r*_S_ = 0.8, vAI: *r*_S_ = 0.7, vDiff: *r*_S_ = 0.7, vDiff/vTFS-Ratio: *r*_S_ = 0.7, *p* < 0.05, two-sided).

In VM, correlations were less pronounced when considering the semi-quantitative visual grading parameters (cochlea, inner ear) and most prominent for the vestibulum (vELS/vTFS-Ratio: *r*_S_ = 0.5, *p* < 0.05, two-sided). Here, age (ELS mean: *r*_S_ = 0.6, ELS ipsilateral: *r*_S_ = 0.6, ELS/TFS ipsilateral: *r*_S_ = 0.7, ELS/TFS: *r*_S_ = 0.7, vELS mean: *r*_S_ = 0.7, vELS ipsilateral: *r*_S_ = 0.6, vELS/vTFS: *r*_S_ = 0.7, vAI: *r*_S_ = 0.5, cDiff: *r*_S_ = 0.5, cDiff/cTFS-Ratio: *r*_S_ = 0.5, *p* < 0.05, two-sided) and age when dizziness started (ELS/TFS: *r*_S_ = 0.5, AI: *r*_S_ = 0.7, Diff: *r*_S_ = 0.6, Diff/TFS-Ratio: *r*_S_ = 0.5, vELS/vTFS: *r*_S_ = 0.5, vELS/vTFS ipsilateral: *r*_S_ = 0.5, vAI: *r*_S_ = 0.6, *p* < 0.05, two-sided) correlated with quantitative ELS measurements. ELS parameters of the cochlea correlated with mean PTA threshold (cELS/cTFS-Ratio, *r*_S_ = 0.6, *p* < 0.05, two-sided). In definite VM, ELS parameters also correlated to the mean value of VOG during caloric irrigation (cAI: *r*_S_ = 0.6, cDiff: *r*_S_ = 0.6, *p* < 0.05 two-sided) and mean HIT (vAI: *r*_S_ = 0.6, vDiff: *r*_S_ = 0.6, vDiff/vTFS: *r*_S_ = 0.6, *p* < 0.05, two-sided).

In VM-MD, cochlear volumetric symmetry proxies (cDiff; cDiff/cTFS) mostly correlated with visual grading asymmetry-indices (AI: *r*_S_ = 0.5, cAI: *r*_S_ = 0.5, *p* < 0.05, two-sided) and grades (cochlea: *p* < 0.05; inner ear: *p* < 0.05). Cochlear AI correlated with PTA variables (mean: *r*_S_ = 0.7, ipsilateral: *r*_S_ = 0.8, when initial PTA > 25 dB: *r*_S_ = 0.9, *p* < 0.05, two-sided). Furthermore, ELS quantification parameters correlated with ipsilateral HIT gain (AI: *r*_S_ = 0.8, *p* < 0.05, two-sided).

**Figure 3:**
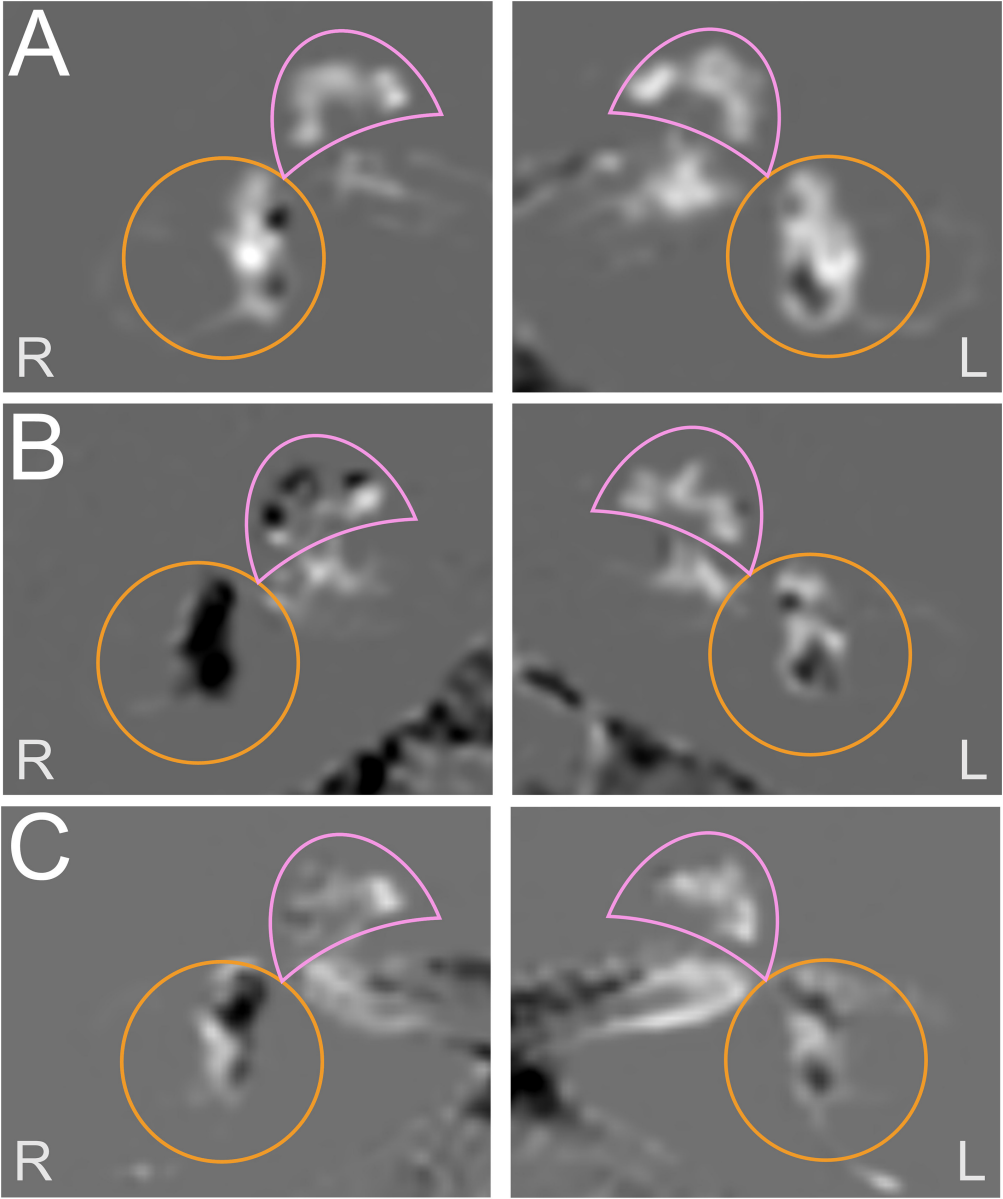
Exemplary cases studies in each a VM, MD, and VM-MD patient. Each row depicts the left (L) and right I side of an exemplary HYDROPS-Mi2 single case image of each a VM **(A)**, MD **(B)**, and VM-MD **(C)** patient. In these images, the endolymphatic space (ELS) presents dark and the Gadolinium-enhanced perilymphatic space (PLS) as well as the bony labyrinth bright white. The cochlea is highlighted with a pink semicircle and the vestibulum with an orange circle. The neurophysiological results (PTA, vHIT waveforms, cVOG data, o/cVEMP waveforms) of all three cases are shown in the Supplementary Figures 1–3. The upper row depicts an exemplary VM single case **(A)**. The 53-year-old female was diagnosed with definite VM and reported migrainous headache with phono- and photophobia, nausea, vomiting, no ear symptoms, and no deficits in the neurotological testing results (PTA, bithermal caloric test, vHIT) (Supplementary Figure 1). The HYDROPS-Mi2 images of the left (L) and right side (R) show no endolymphatic hydrops (EH) on both sides following the classification of Nakashima et al. (21). The middle row shows an exemplary MD single case **(B)**. The 69-year-old male patient with definite MD exhibited profound hearing loss and aural fullness on the right ear during an attack and significant ipsilateral auditory (right-sided initial PTA of 36 dB with low tone hearing loss pattern) and ipsilateral vestibular dysfunction (>30% side difference in the caloric test; normal vHIT gain; oVEMP not reproducible on the right, cVEMP asymmetry ratio of 10% disadvantage of the right) immediately after an attack (Supplementary Figure 2). The HYDROPS-Mi2 images of the right inner ear show severe EH (grade 2) in both the cochlea and the vestibulum. There is no EH (grade 0) in the left inner ear. The lower row shows an exemplary VM-MD single case I. The 72-year old female patient with a constellation of clinical symptoms and diagnostic findings fit both definite VM and MD (VM-MD). During an attack, she described migrainous headache with phono- and photophobia, nausea, as well as bilateral ear symptoms (aural fullness, tinnitus). Neurophysiological testing revealed a right-sided auditory (PTA of 90 dB with low tone hearing loss pattern) and no pathological side difference in the caloric test, vHIT, or o/cVEMPs (Supplementary Figure 3). The HYDROPS-Mi2 images of the right inner ear show a mild EH (grade 1) in the cochlea and the vestibulum and no EH (grade 0) in the left inner ear.

Figure 3 shows each a typical VM, MD, and VM-MD case, including their imaging (Figure 3) results. Neurophysiological results (PTA, vHIT waveforms, cVOG data, o/cVEMP waveforms) of all three cases are shown in the Supplementary Material.

## Discussion

The current study focused on EH characteristics (extent and location) and its relationship with neurophysiological results in patients with VM and MD and patients who fulfilled the diagnostic criteria for both diseases (VM-MD). EH was quantified visually following the classification of Nakashima et al. (21) and using volumetric local thresholding (VOLT) assessment implemented for HYDROPS-Mi2 images. As a result, EH was only present in a small proportion of VM patients (both with auditory symptoms) but frequently found in MD patients (Tables 1, 2). Location and laterality of EH and neurophysiological testing classifications were positively associated (Table 3). In MD, visual semi-quantitative grading and volumetric quantification correlated highly to each other, as well as side differences in VOG during caloric irrigation and low tone hearing loss patterns in PTA. In VM, correlations were less pronounced and were also found between cochlear volumetric quantification parameters and PTA. VM-MD assumed an intermediate position between VM and MD.

To begin, the findings support some overlap of MD and VM (1, 31) based on the current diagnostic criteria. Hence, EH could be considered as inner ear damage due to different etiologies. The current diagnostic criteria recommend that whenever patients fulfill the criteria for both VM and MD – especially hearing loss documented by audiometry – MD should be diagnosed, even if migraine symptoms occur during the vestibular and auditory attacks (1). In contrast, the data show that EH's morphological evidence was present in patients with auditory symptoms regardless of whether they meet MD or VM criteria. VM patients who were mostly free from auditory symptoms did not show any morphological evidence of EH. Thus, a future revision of the official classification might consider patients with overlapping audio-vestibular dysfunction as a VM-MD overlap syndrome. EH's current anatomical evidence suggests a joint dysfunction of the inner ear rather than differential physiological mechanisms between the two disorders (3, 32). Unlike previous studies (33–37), no significant o/cVEMP differences were found between VM, MD, and VM-MD.

Evidence continues to emerge, suggesting common contributing factors in both diseases, VM and MD, such as inner ear vasospasm or vasculopathy, neuropeptide derangements, hormonal interactions, calcium channelopathies, or disturbance of salt metabolism (38–40). During a VM episode, a subpopulation of serotonergic and non-serotonergic dorsal raphe nucleus cells may comodulate the processing in the vestibular nuclei and the central amygdaloid nucleus, which also activates the central vestibular processing pathway and contributes to VM (41). It was shown that the trigeminal nerve could play an essential role in the development of VM attacks because it provides a dense sensory innervation of cerebral, basilar and meningeal blood vessels and thus of the inner ear arteries via the anterior inferior cerebellar artery (AICA). Indeed, it has been shown that the cochlea and the vestibular labyrinth receive trigeminal innervation via the ophthalmic branch that provides parasympathetic innervation to the basilar artery and the AICA (42). Activation of perivascular trigeminal nerve endings causes the release of substance P and calcitonin gene-related peptide (CGRP), leading to local neuroinflammation, permeability changes, vasodilatation, and edema (43, 44). Thus, the trigeminal nerve directly affects neuroinflammation and blood flow of the inner ear, supporting the notion that neurovascular effects of migraine can affect inner ear structures (42, 45–47) and explain symptoms and EH.

Moreover, chronically fluctuating hydropic inner ears are less able to auto-regulate their vasculature against the changes induced by acute migraine attacks and may ultimately manifest as persisting EH (47–50). Accordingly, cases have been reported with migraine-associated vascular changes that caused inner ear ischemia and EH, resulting in sensorineural hearing loss and MD-like symptoms (51) or migraine-associated hearing loss with a severe bilateral EH without vertigo (52). Spreading cortical depression or spreading brainstem depression (53) are theorized to cause migraine aura (in this case, vertigo, dizziness, and auditory dysfunction) and symptoms that ultimately result also in substance P release from the trigeminal ganglion, leading to vasodilation, increased vascular permeability, and extravasation of plasma and neurogenic inflammation (54–56).

Although MD and VM's pathophysiological relationship is so far unclear (22), some researchers believe that the two disorders may represent a continuum syndrome rather than existing as separate conditions. VM could be caused primarily by an abnormality of the central and peripheral vestibular pathways, whereas MD could mainly be associated with peripheral membranous labyrinthine dysfunction. The auditory symptoms in VM patients may be related to vasospasm- and hypoperfusion-induced ischemia of labyrinthine structures or serotonergic-induced extravasation (57). Histopathology has provided evidence that not every individual with EH presents with MD symptoms (58, 59), and not every individual with the clinical diagnosis of MD has EH (60), which is also the case in the current data. Earlier studies found EH in 60–90% of MD patients and EH's presence in asymptomatic contralateral sides in 65% of MD patients (61). Thus, there seems to be no match between the occurrence of EH and normal neurophysiological values. This could point to MD being a systemic disease, which has been argued before and should be further elucidated. The development of inner ear imaging allows EH to be demonstrated in a human *in vivo* and provides the basis for quantifying the endolymphatic space and its dilatation within a single imaging sequence (21, 61). Since it has been shown that EH can differently affect cochlear and vestibular compartments of the inner ear in patients with VM (62, 63), EH imaging has been used in the differential diagnosis of patients with suspected MD or VM (64). The finding that VM patients are only associated with EH when showing auditory symptoms suggests an MD-like pathology.

As a consequence, EH seems to point toward an inner ear pathology due to different etiologies. Fittingly, EH extent correlated with auditory and vestibular testing. However, EH was also observed in asymptomatic individuals (61) and post-mortem in individuals' temporal bone without ear diseases (65). Besides, the complaints reported by patients with VM and MD overlapped frequently. Therefore, some authors suggest that future diagnostic criteria should be based on pathophysiology and not only on symptoms (66).

### Methodical Limitations

There are methodical limitations in the current study that need to be considered in the interpretation of the data. Although this study has a relatively high number of subjects per pathology, a significantly higher number of participants would likely show more robust results, especially given the division into definite and probable patient groups or the small group of diagnostic overlaps (VM-MD). Second, the persistence of EH is yet under discussion. There is evidence that it may fluctuate depending on the attack (VM) or become more likely depending on the disease's duration (MD). Accordingly, the present results should be treated with caution. Third, the statistical analysis suffers from a parallel evaluation of the categorical and scalar values. It would be desirable to develop a statistical model for both types of information. Fourth, the lack of selectivity of the classifications criticized in the study could also affect the statements made here. A study to examine the discriminatory power of symptoms and diagnostic results between VM and MD diagnoses would be crucial for future claims.

## Conclusion

The current study on MRI imaging of EH has shown that (1) a cochlear and vestibular hydrops can occur in MD and VM patients with auditory symptoms; this suggests inner ear damage irrespective of MD diagnosis or VM. (2) The EH grades often correlated with auditory symptoms such as hearing impairment and tinnitus. (3) Further research is required to uncover whether migraine is one causative factor of inner ear pathology that leads to a common final pathway of EH or whether EH in VM patients with auditory symptoms suggests an additional pathology due to MD.

## Data Availability Statement

The original contributions presented in the study are included in the article/Supplementary Material, further inquiries can be directed to the corresponding author/s.

## Ethics Statement

The studies involving human participants were reviewed and approved by Institutional Review Board at Jeonbuk National University Hospital. The patients/participants provided their written informed consent to participate in this study.

## Author Contributions

S-YO, VK, and MD designed and directed the project. BL and J-JK collected the data and wrote the manuscript with support from S-YO and N-RL. S-BH, RB, and JG verified the analytical methods and analyzed the image data. All authors contributed to the article and approved the submitted version.

## Conflict of Interest

The authors declare that the research was conducted in the absence of any commercial or financial relationships that could be construed as a potential conflict of interest.

## Supplementary Material

The following figures show the neurophysiological results of the exemplary case studies in each patient with vestibular migraine (VM, Supplementary Figure 1), Meniére's disease (MD, Supplementary Figure 2), or with both disease characteristics (VM-MD, Supplementary Figure 3). The results consist of pure tone audiograms (PTA, **A**), video-oculography with response waveforms after caloric irrigation. **(B)** Head impulse test waveforms **(C)**, cervical and ocular vestibular evoked myogenic potentials (c/oVEMP, **D**, **E**) for the left (L) and right (R) side, respectively. “L/R” in **(D)** (cVEMP) means the response of the left/right sternocleidomastoid muscle (SCM) during the stimulation on the ipsilateral left/right ear. “L/R” in **(E)** (oVEMP) means the left/right stimulating ear with recordings on the contralateral extraocular muscles.

Supplementary Figure 1 Neurophysiological results for the exemplary VM case. The 53-year-old female was diagnosed with definite VM and reported migrainous headache with phono- and photophobia, nausea, vomiting, no ear symptoms. Fittingly the neurotological testing results show no deficits (PTA, bithermal caloric response, vHIT, c/oVEMPs).

Supplementary Figure 2 Neurophysiological results for the exemplary MD case. The 69-year-old male patient with definite MD exhibited profound hearing loss and aural fullness on the right ear during an attack and significant ipsilateral auditory (right-sided initial PTA of 36 dB with low tone hearing loss pattern) and ipsilateral vestibular dysfunction with >30% side difference in the caloric test. He showed normal vHIT gain and normal cVEMP but increased oVEMP asymmetry ratio of 57% due to increased ipsilateral oVEMP amplitude immediately after an attack.

Supplementary Figure 3 Neurophysiological results for the exemplary VM-MD case. The 72-year old female patient with a constellation of clinical symptoms and diagnostic findings that fit both definite VM and MD (VM-MD). During an attack, she described migrainous headache with phono- and photophobia, nausea, as well as bilateral ear symptoms (aural fullness, tinnitus). Neurophysiological testing revealed a right-sided auditory (PTA of 90 dB with low tone hearing loss pattern) and no pathological side difference in the caloric test, vHIT, or o/cVEMPs.

## Abbreviations

3D: Three-dimensional
3D mean: ELS volume mean of the right and left inner ear
AAO-HNS: American Academy of Otolaryngology-Head and Neck Surgery
AC: Air conducted
AR: Asymmetry ratio
cELS: ELS of the cochlea
cTFS: TFS of the cochlea
cVEMP: cervical vestibular-evoked myogenic potential
dB: Decibel
Diff: The difference in volume between the left and right side
DOF: Degrees of freedom
EH: Endolymphatic hydrops
ELS: Endolymphatic space
FA: Flip angle
FLAIR: Fluid-attenuated inversion recovery
FOV: Field of view
GRAPPA: Generalized auto-calibrating partially parallel acquisition
IRB: Institutional Review Board
HYDROPS: HYbriD of Reversed image Of Positive endolymph signal and native image of positive perilymph Signal
hT2W: heavily T2-weighted
iMRI: Intravenous contrast agent enhanced MRI of the inner ear
L: Left
MD: Meniere's Disease
MRC: MR cisternography
MRI: Magnetic resonance imaging
NEX: Number of excitations
No: Number
oVEMP: ocular vestibular-evoked myogenic potential
PEI: Positive endolymphatic image
PLS: Perilymphatic space
PPI: Positive perilymph image
PTA: Pure tone audiometry
R: Right
SD: Standard deviation
SF: Supplementary figure
SPACE: Sampling perfection with application-optimized contrasts by using different flip angle evolutions
TE: Echo time
TFS: Total fluid space
TR: Repetition time
vHIT: Video head impulse test
VM: Vestibular Migraine
VEMP: Vestibular evoked myogenic potential
VOG: Video-oculography
VOLT: Volumetric Local Thresholding
vELS: ELS of the vestibulum
vTFS: TFS of the vestibulum.
