# Supplementary figures and images for: Endolymphatic Hydrops in Patients With Vestibular Migraine and Concurrent Meniere's Disease
Source: Front Neurol. 2021 Mar 11;12:594481. doi: 10.3389/fneur.2021.594481 (PMC7991602; doi:10.3389/fneur.2021.594481)

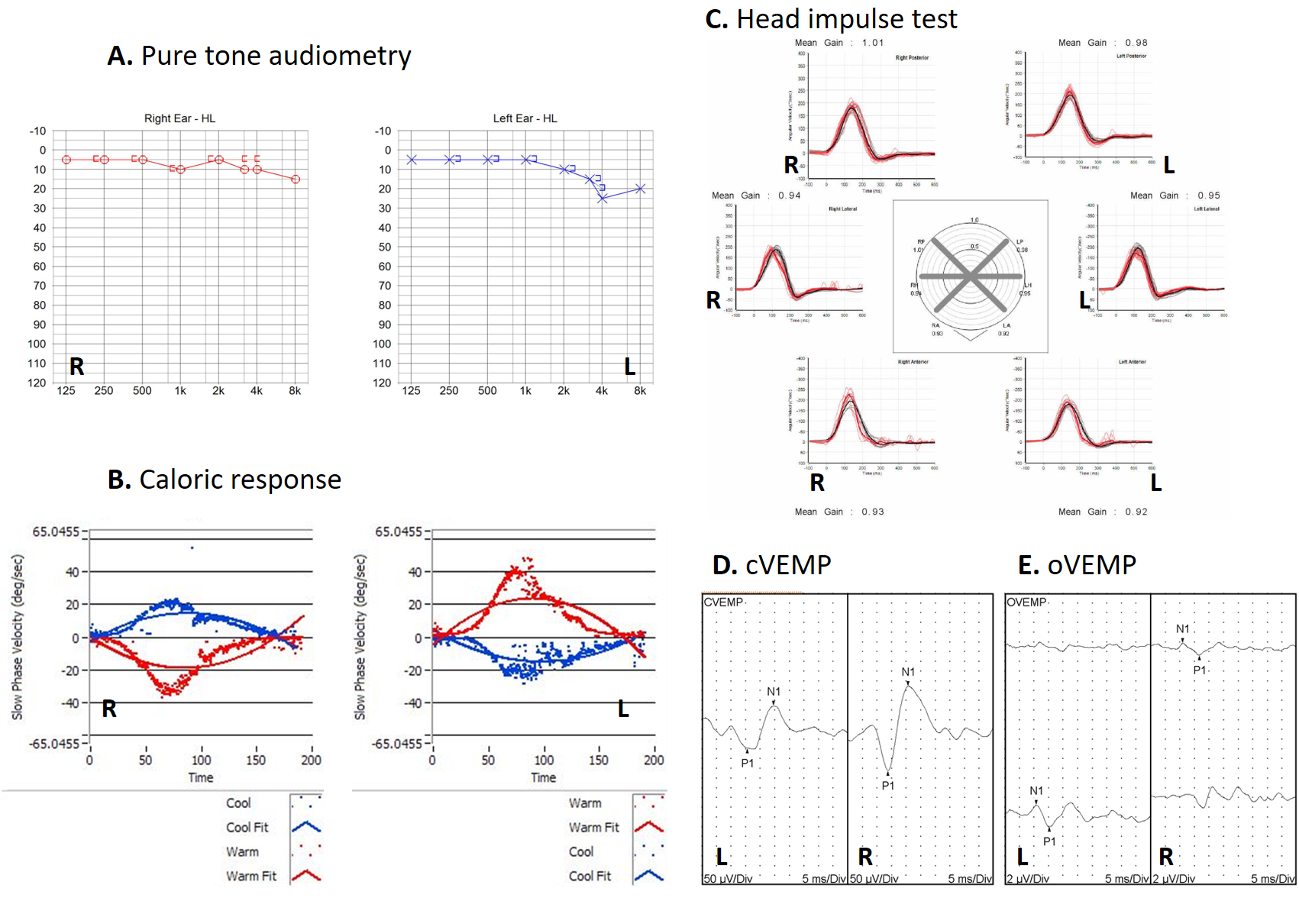

Supplement: Supplementary Figure 1 — Neurophysiological results for the exemplary VM case. The 53-year-old female was diagnosed with definite VM and reported migrainous headache with phono- and photophobia, nausea, vomiting, no ear symptoms. Fittingly the neurotological testing results show no deficits (PTA, bithermal caloric response, vHIT, c/oVEMPs). [file Image_1.TIF]

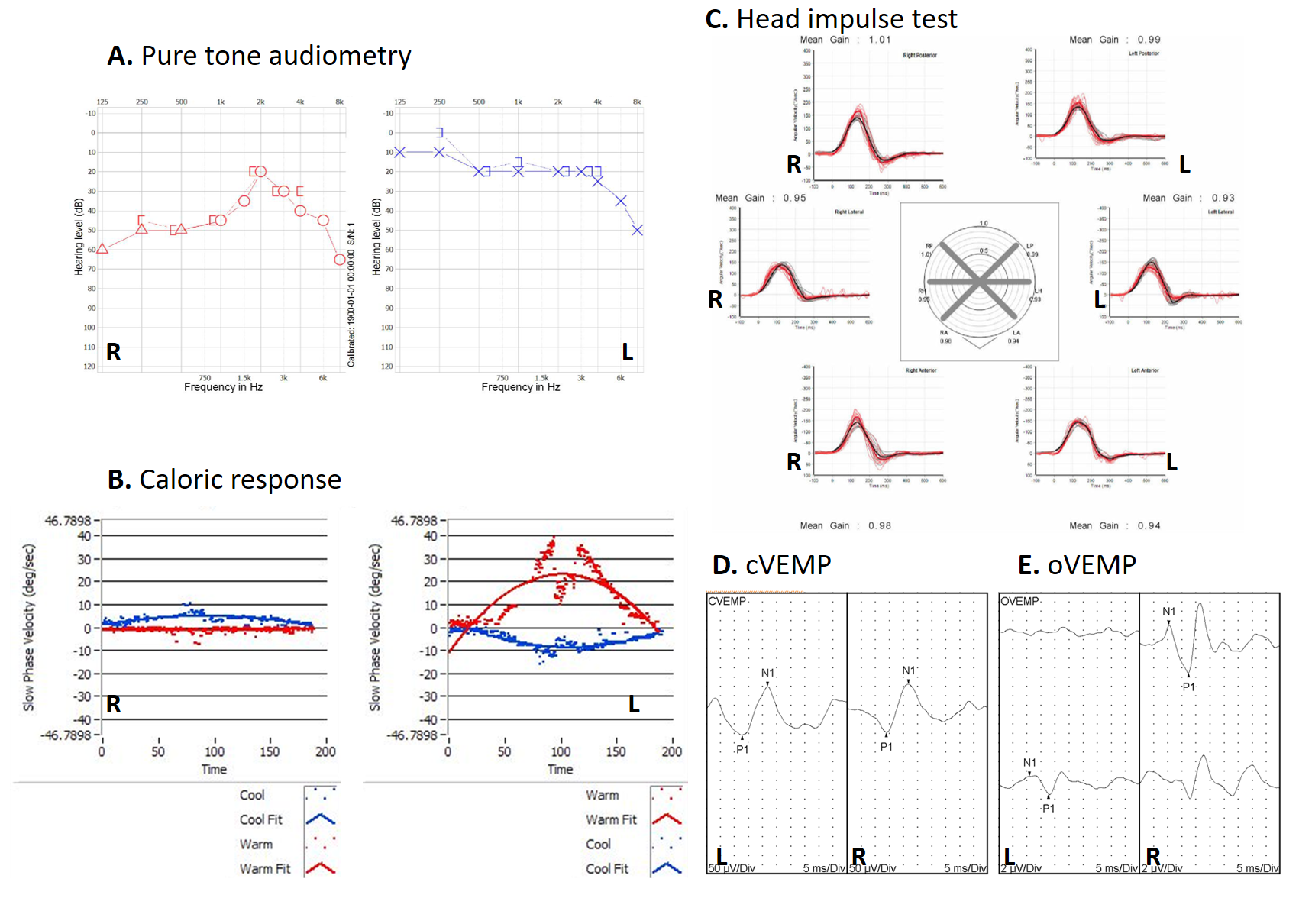

Supplement: Supplementary Figure 2 — Neurophysiological results for the exemplary MD case. The 69-year-old male patient with definite MD exhibited profound hearing loss and aural fullness on the right ear during an attack and significant ipsilateral auditory (right-sided initial PTA of 36 dB with low tone hearing loss pattern) and ipsilateral vestibular dysfunction with >30% side difference in the caloric test. He showed normal vHIT gain and normal cVEMP but increased oVEMP asymmetry ratio of 57% due to increased ipsilateral oVEMP amplitude immediately after an attack. [file Image_2.TIF]

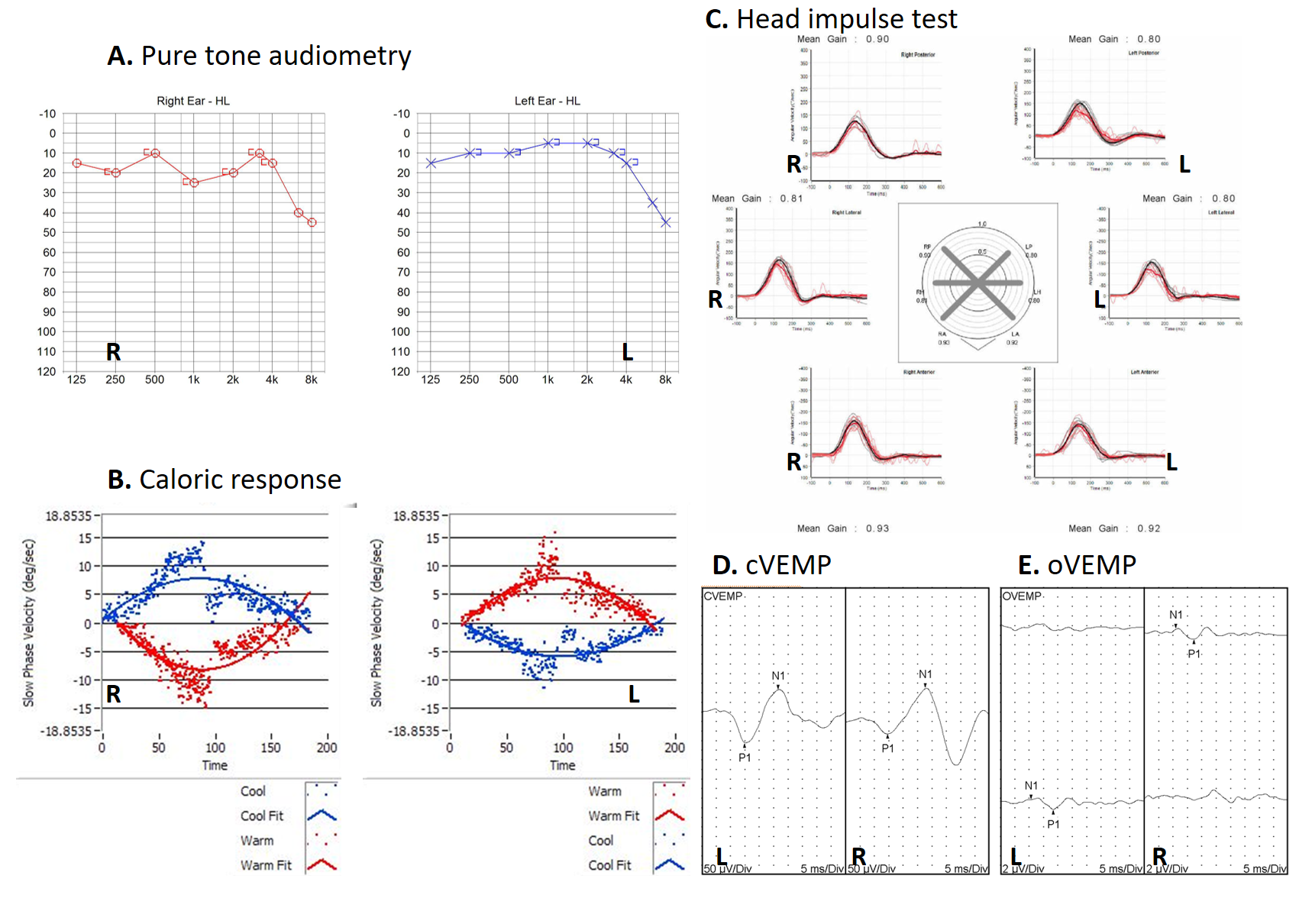

Supplement: Supplementary Figure 3 — Neurophysiological results for the exemplary VM-MD case. The 72-year old female patient with a constellation of clinical symptoms and diagnostic findings that fit both definite VM and MD (VM-MD). During an attack, she described migrainous headache with phono- and photophobia, nausea, as well as bilateral ear symptoms (aural fullness, tinnitus). Neurophysiological testing revealed a right-sided auditory (PTA of 90 dB with low tone hearing loss pattern) and no pathological side difference in the caloric test, vHIT, or o/cVEMPs. [file Image_3.TIF]
